# AI-Based Automated Lipomatous Tumor Segmentation in MR Images: Ensemble Solution to Heterogeneous Data

**DOI:** 10.1007/s10278-023-00785-1

**Published:** 2023-02-28

**Authors:** Chih-Chieh Liu, Yasser G. Abdelhafez, S. Paran Yap, Francesco Acquafredda, Silvia Schirò, Andrew L. Wong, Dani Sarohia, Cyrus Bateni, Morgan A. Darrow, Michele Guindani, Sonia Lee, Michelle Zhang, Ahmed W. Moawad, Quinn Kwan-Tai Ng, Layla Shere, Khaled M. Elsayes, Roberto Maroldi, Thomas M. Link, Lorenzo Nardo, Jinyi Qi

**Affiliations:** 1 Department of Biomedical Engineering, University of California, Davis, CA USA; 2 Department of Radiology, UC Davis Health, Sacramento, CA USA; 3 Radiotherapy and Nuclear Medicine Department, South Egypt Cancer Institute, Assiut University, Assiut, Egypt; 4 Department of Radiology ASST Spedali Civili, Brescia, Italy; 5 Section of Radiology, Department of Medicine and Surgery (DiMeC), University of Parma, Parma, Italy; 6 Pathology and Laboratory Medicine, University of California Davis, Sacramento, CA USA; 7 Department of Biostatistics, Fielding School of Public Health, University of California, Los Angeles, CA USA; 8 Department of Radiological Sciences, University of California, Irvine, CA USA; 9 Department of Diagnostic Radiology, McGill University Health Center, Montreal, Canada; 10 Department of Diagnostic Imaging, University of Texas MD Anderson Cancer Center, Houston, TX USA; 11 Department of Diagnostic Radiology, Mercy Catholic Medical Center, Darby, PA USA; 12 Department of Radiology and Biomedical Imaging, University of California, San Francisco, CA USA

## Abstract

Deep learning (DL) has been proposed to automate image segmentation and provide accuracy, consistency, and efficiency. Accurate segmentation of lipomatous tumors (LTs) is critical for correct tumor radiomics analysis and localization. The major challenge of this task is data heterogeneity, including tumor morphological characteristics and multicenter scanning protocols. To mitigate the issue, we aimed to develop a DL-based Super Learner (SL) ensemble framework with different data correction and normalization methods. Pathologically proven LTs on pre-operative T1-weighted/proton-density MR images of 185 patients were manually segmented. The LTs were categorized by tumor locations as distal upper limb (DUL), distal lower limb (DLL), proximal upper limb (PUL), proximal lower limb (PLL), or Trunk (T) and grouped by 80%/9%/11% for training, validation and testing. Six configurations of correction/normalization were applied to data for fivefold-cross-validation trainings, resulting in 30 base learners (BLs). A SL was obtained from the BLs by optimizing SL weights. The performance was evaluated by dice-similarity-coefficient (DSC), sensitivity, specificity, and Hausdorff distance (HD95). For predictions of the BLs, the average DSC, sensitivity, and specificity from the testing data were 0.72 $\pm$ 0.16, 0.73 $\pm$ 0.168, and 0.99 $\pm$ 0.012, respectively, while for SL predictions were 0.80 $\pm$ 0.184, 0.78 $\pm$ 0.193, and 1.00 $\pm$ 0.010. The average HD95 of the BLs were 11.5 (DUL), 23.2 (DLL), 25.9 (PUL), 32.1 (PLL), and 47.9 (T) mm, whereas of SL were 1.7, 8.4, 15.9, 2.2, and 36.6 mm, respectively. The proposed method could improve the segmentation accuracy and mitigate the performance instability and data heterogeneity aiding the differential diagnosis of LTs in real clinical situations.

**Supplementary Information:**

The online version contains supplementary material available at 10.1007/s10278-023-00785-1.

## Introduction

Lipomatous tumors (LTs) refer to a broad spectrum of mesenchymal tumors that range from benign entities such as lipoma to aggressive malignant tumor including liposarcoma [1]. Magnetic resonance imaging (MRI) is the modality of choice for evaluating LTs for its superior soft tissue contrast. It is used to distinguish benign lipomas from their malignant mimics, the well-differentiated liposarcomas. To distinguish these lesions, radiologists rely on visual evaluation of multiple MRI features including the level of fat saturation, architectural complexity in relation to the normal surrounding adipose tissue and pattern of contrast enhancement [2]. However, the qualitative assessment has demonstrated variable levels of diagnostic performance with reported accuracy levels ranging from 58 to 74% [3–5]. MR exams are often composed of hundreds or even thousands of images, and subtle important image features may be missed since interpretation itself is a complex visual search process that relies on several cognitive processes, including selective attention, working memory, and decision-making [6]. Diagnostic aids from quantitative radiomics have shown encouraging results in the characterization of many diseases including diagnosis of myocardial infarction [7] and small vessel disease [8], quantification of liver fibrosis [9], and classification [10] and grading [11] of soft tissue tumors. In LTs, 73–99% accuracy has been reported [12–20] for differentiating malignant from benign lesions. However, extraction of radiomic features is challenging because the identification of the tumor extent and its segmentation are labor-intensive and prone to human error, especially given that many of these tumors insinuate between soft tissue planes and can exceed 10 cm in their longest dimension [21]. In our experience, manual delineation of LTs requires not only well-trained experts, but also is time-consuming. For example, segmenting one tumor could take 4 h or even more. Furthermore, there is no generalized prior knowledge of LTs that could be used in an atlas-based method, which is frequently used in neuroscience studies.

Deep learning (DL) has been proposed to automate medical image segmentation, which subsequently can be used in multiple image analysis domains including improved radiological diagnostics [22, 23]. Unlike organ segmentation having certain prior information, automatically segmenting LTs in MR images poses a great challenge from biological and scanner-related perspectives. LTs are biologically heterogeneous and can be strikingly indistinguishable compared to the surrounding normal adipose tissue or may vary significantly from surrounding tissue. Also, they may arise anywhere in the body, which widens the complexity of anatomical shape analysis. Furthermore, MRI images may harbor scanner hardware and software related artifact, such as signal intensity inhomogeneity from surface coils. The range and meaning of MRI image intensity values vary for the same protocol and even for the same body region [24]. Additional sources of MR data heterogeneity include the employment of numerous acquisition and processing protocols for the same clinical application both within and in-between different institutions and the acquisition of variable sizes of field-of-view (FOV) with subsequent voxel size variability. Although the resulting heterogeneous data do not necessarily impair the physicians’ qualitative diagnostic output, it poses a significant challenge for any pre-processing algorithm attempting to achieve high segmentation accuracy and robust stability using deep learning [25]. These factors mentioned above increase the performance instability of supervised trainings and decrease the generalizability of the selected training data distribution to new data. Despite better data quantity requirement for training a neural network in this study, integrating data from multiple sources has ramifications, and it is not easy to find a solution in a single network design. To mitigate the data heterogeneity problem, we proposed an ensemble learning framework combining multiple convolutional neural networks (CNNs) trained individually using K-fold cross-validation with the training data curated using a variety of correction and normalization methods. Some features of the heterogeneous training data might be missing when using only one data preparation method and result in poor segmentation performance. The complimentary features might be presented by different data preparation methods to the neural networks, and the ensemble learning framework could provide better segmentation performance by optimally weighting all the trained neural networks for testing data instead of equal-weighted averaging. The aim of this work was to study the segmentation accuracy and stability of our proposed DL-based Super Learner approach for delineating LTs on T1-weighted (T1W) or proton-density (PD) MR images, curated from multiple centers, in comparison to any single trained neural network.

## Materials and Methods

### I. MR Images and Training Data Preparation

This study used non-fat saturated T1W or PD MR images from 185 patients; 83 males, 102 females; age 58.4 ± 11.9 years (range: 23–89 years), who underwent musculoskeletal MRI as part of the pre-operative evaluation of their LTs at 4 participating institutions. Details on cohort identification are similar to the ones that have been previously described [26]. The study was approved by the Institutional Review Board at the host university, and several data transfer agreements were in effect between host university and other universities. All the procedures were HIPAA-compliant, and an informed consent was waived for this retrospective chart review study at all sites. All patients underwent surgical resection of the tumor within 3 months from the date of their MRI scans, and histopathology was available for all the resected lesions. MRI data were acquired using several different MR scanners similarly as previously described [26], all studies included at least acquisition of T1W or PD sequence. A fellowship-trained, board-certified radiologist with 3 year-experience after musculoskeletal fellowship (LN) reviewed MR images and categorized the anatomical location of the tumor into 5 groups: distal upper limb (DUL, *n* = 5), distal lower limb (DLL, *n* = 3), proximal upper limb (PUL, *n* = 41), proximal lower limb (PLL, *n* = 88) or Trunk (T, *n* = 48, which also included LTs in the neck region). Imbalance in tumor location is mandated by the characteristics of the studied disease, and these custom tumor categories were introduced to be used for the systematic analysis in this work, despite no general guideline for categorization yet being proposed.

Anonymous MRI images in DICOM format were imported into a viewing workstation running OsiriX MD v.9.5.1. (Pixmeo, Switzerland). Two radiology residents (both at their 4th year of training [PGY-4]) reviewed the images, identified the tumor on the described MR sequence and delineated the tumor manually slice-by-slice on the axial plane. All delineations were checked for accuracy by a third resident (PGY-4), and any required modifications were implemented. The residents were closely supervised by a board-certified radiologist while delineating the tumors. The delineations were randomly selected for validation from different residents. The final delineations were exported as training masks (ground truths) in DICOM format. For each series, a corresponding mask series was created, with intensity of 1 representing tumor and 0 representing the background.

All the MR images and their masks were converted from DICOM format to raw data format (float 32-bit) and resampled to the image matrix size of 512 × 512 with the corresponding voxel size while preserving the aspect ratio (mostly from higher dimensions). The different scanners and acquisition protocols across the participating institutions can result in signal intensity range variation and non-uniformity (e.g., due to inhomogeneous magnetic field, instable gradient field, susceptibility and different surface coils, and non-specific relationship between signal intensity and tissue types). The N4ITK approach (N4 correction) [27] was proposed to correct inhomogeneous magnetic field (bias field) in MR images. To improve the stability and accuracy of the LT segmentation by taking advantage of the ensemble learning, multiple neural networks were trained with the data undergoing different pre-processing methods. In this work, four normalization/standardization methods were individually investigated and combined with the N4 correction: Z-score (mean and standard deviation), max/min per three-dimensional tumor volume, max/min per slice, and Nyul standardization [28] methods. Each normalization or standardization method could suppress certain features of the MR images due to pixel value scaling or compression.

The 185 patient exams were single split for fivefold cross-validation trainings (80%, 148 exams), optimization of the ensemble weight (9%, 17 exams), and final performance testing (11%, 20 exams). For the fivefold cross-validation trainings (base learners), the 148 exams were randomly split into five equal groups, and each group was used as validation data in turn and the rest as training data. The training data were augmented on-the-fly by using rotation, scaling, and flipping horizontally and vertically. To estimate the ensemble weights of the base learners in the ensemble network, 17 exams were used in the calculation of the super learning loss. The final performance was evaluated using the last 20 exams for each base learner and the super learner.

### II. CNN Architectures

**Fig. 1:**
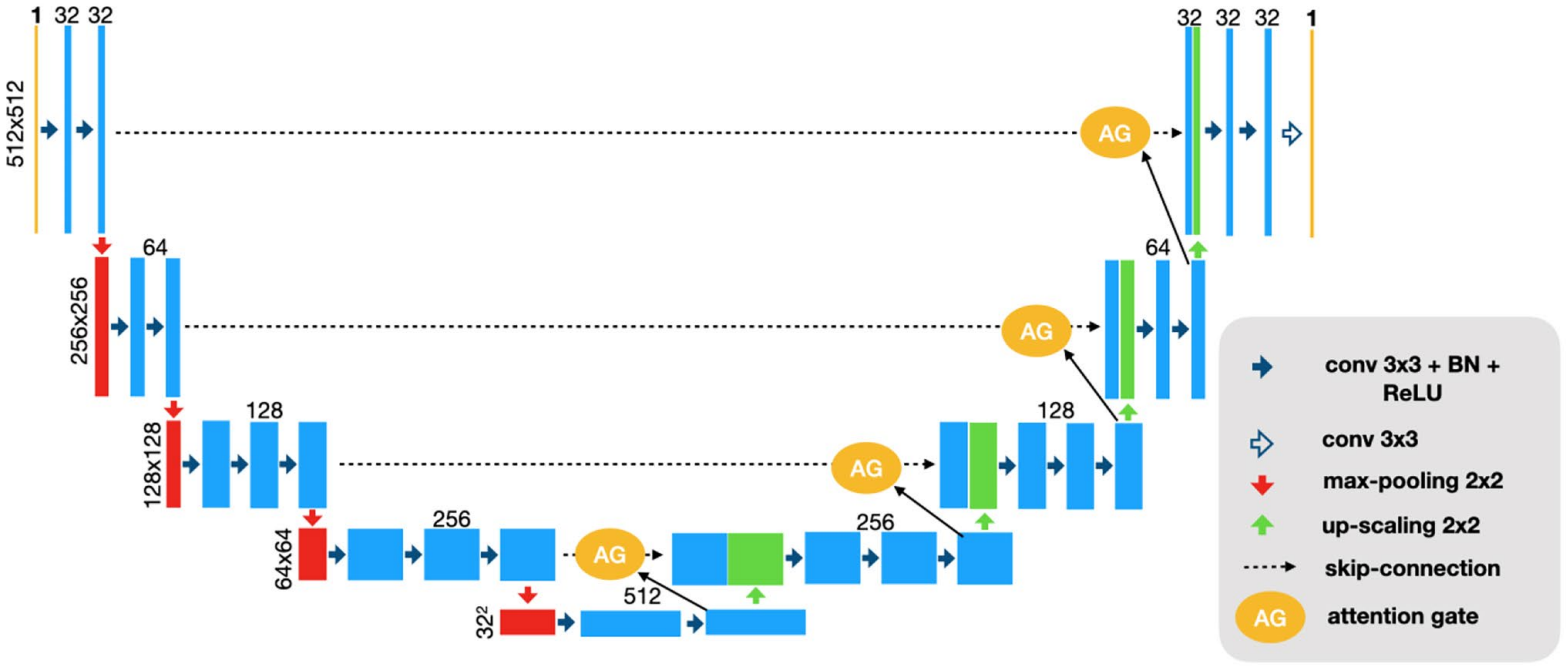
The U-Net architecture with the optional attention gate (AG). The feature maps from the skip-connections were scaled using the attention coefficients calculated in attention gates with the feature maps from the coarser level. Both networks with and without the attention gates were implemented in this work

The U-Net architecture is known for its superiority in multi-resolution feature extraction. A standard U-Net [29] and attention U-Net [30] were implemented using TensorFlow as shown in Fig. 1. The U-Net was composed of five resolution levels of convolutional layers, and each level was skip-connected between the encoding and decoding processes. The root feature of the convolutional layers on the first level was 32 and increased by a factor of 2 every level down. Each convolutional layer was followed by a BatchNorm layer [31] and a Rectified Linear Unit (ReLU) activation function. The sigmoid activation function was used in the final layer to output a probability map for tumor segmentation. The attention U-Net was implemented on top of the U-Net with additional attention blocks in the skip-connections to focus on extracting self-attention features in the region-of-interest. As a result, there are 12.5 and 13.6 million trainable parameters in the U-Net and attention U-Net, respectively.

### III. CNN Training and Ensemble Learning

The U-Nets were individually trained with the MR images normalized by Z-score before and after the N4 correction and by the other three normalization methods after the N4 correction, whereas the attention U-Net was trained with the images normalized by Z-score after the N4 correction. Since the MR images are too divergent to be completely corrected for the bias field by the N4 correction without losing some feature information, we decided to train one of the U-Nets with the data before the correction. A dice-coefficient loss function was optimized by Adam optimizer [32] to train the individual base learners, U-Nets, and attention U-Nets. The batch size of 16 image slices and total epoch number of 120 were used for all the trainings of the base learners.

**Fig. 2:**
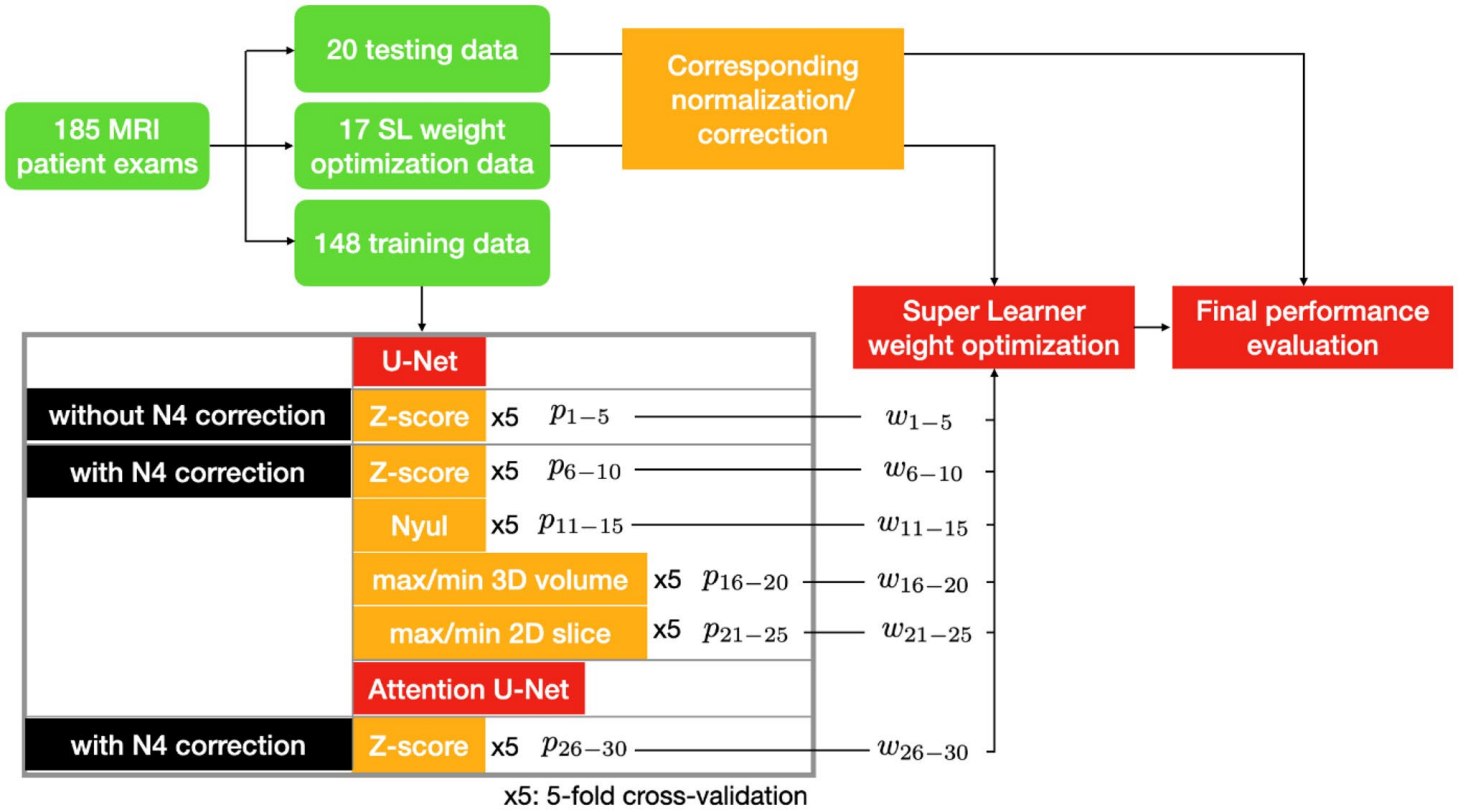
The workflow of the ensemble framework. The curated patient exams were split into three groups for the three steps: neural network training, super learner weight optimization, and final performance evaluation. All the exams first underwent the corresponding normalization/correction. Each neural network was trained using fivefold cross-validation to obtain the base learners, ${p}_{j}$*,* whose super learner weights, ${w}_{j}$, were optimized before being used in the final performance evaluation

After reviewing the performance of the base learners, not a single trained base learner or image normalization method in this work could always result in good segmentation performance for any specific patient data because of the data heterogeneity. In addition, the stability of the optimization in the trainings could vary a lot due to the representativity of the random split training data to the validation and testing data. Therefore, combining all the trained base learners using equal-weighted averaging would not provide optimal results. To mitigate these issues, all the trained base learners were weighted differently and combined using the Super Learner (SL) ensemble framework [33]. The workflow of our proposed ensemble framework is illustrated in Fig. 2.

Before obtaining the SL predicted segmentations of the testing data, the ensemble weights were first optimized by minimizing the following SL loss function:

$${Loss}_{SL}\left(\overrightarrow{w}\right)=-\sum_{i}\left[{y}_{i}log\left(\sum_{j=1}^{m}{w}_{j}{p}_{ji}\right)+\left(1-{y}_{i}\right)log\left(1-\sum_{j=1}^{m}{w}_{j}{p}_{ji}\right)\right],$$

where $i$ is the index of the input image slices, $j$ is the index of the base learners, ${w}_{j}$ is the ensemble weight of the $j$-th base learner, $m$ is the total number of the base learners, ${p}_{ji}$ is the segmentation of the image slice $i$ predicted by the $j$-th base learner, and ${y}_{i}$ is the ground truth (manual delineation) of the image slice $i$. During the optimization, each ensemble weight was restricted to the range from 0 to 1, and the sum of the all ensemble weights was to 1.

The SL predicted probability map, ${p}_{SL,i}$, is computed using the optimized ensemble weights and the corresponding base learners by

$${p}_{SL,i}={\sum }_{j=1}^{m}{w}_{j}{p}_{ji}.$$

A standard threshold of 0.5 was used on the SL predicted probability map to generate the final segmentation. A receiver operating characteristic (ROC) analysis was conducted to study the discriminant capability of the SL and the influence of the threshold on the accuracy of the final segmentation.

In this work, a total of 30 base learners resulted from 6 combinations of the CNNs and correction/normalization methods for fivefold cross-validation trainings as described above. The segmentations of the 17 patient exams were first predicted using these 30 base learners and then fed into the SL loss function as ${p}_{ji}$. The ensemble weights were initialized as 1/30 and optimized using Adam optimizer. It took 75 epochs for the ensemble weights to converge in this work.

The fivefold cross-validation trainings, the ensemble weight optimization and SL prediction were conducted on a NVIDIA Tesla V100 32 GB GPU. The computation time for the cross-validation trainings was approximately 193 s/epoch, for ensemble weight optimization 6 s/epoch and for prediction 19 ms per image slice.

### V. Evaluation Metrics

The performance of the final SL predicted segmentations of the 20 testing data was evaluated against the manual delineations by dice-similarity-coefficient (DSC), sensitivity, specificity, and 95th percentile of the Hausdorff distance (HD95) [34] in comparison with the predictions from the 30 base learners. All the metrics are overlap-based except for HD95 which is boundary distance-based and measures the 95th percentile of the Euclidean distances of the nearest points between the predictions and ground truths. The disadvantage of the overlap-based metrics is that they do not account for the spatial distribution of the correct or misclassified voxels [35]. On the contrary, HD95 is more sensitive to the differences in the delineation contour and spatial position of the voxels. For easier comparison between the SL and base learners, the overlap-based metrics difference was presented directly, since the metrics range is between 0 and 1, while the improvement for HD95 was represented by percentage because the boundary distance is arbitrary depending on the accuracy of the predicted segmentation.

To statistically compare the performance of the individual base learners and SL ensemble predictions for the 20 testing data, we first conducted an ANOVA with post hoc Tukey multiple-comparison adjustment. After removing two significantly underperforming outlier base learners, the average performance of remaining base learners was used for comparisons against SL ensemble predictions. The values of the metrics used for comparing performance (DSC, sensitivity, and specificity) demonstrated skewed distributions; accordingly, the non-parametric equivalent of paired *t* test (i.e., Wilcoxon signed rank test) was used for this comparison.

## Results

**Fig. 3:**
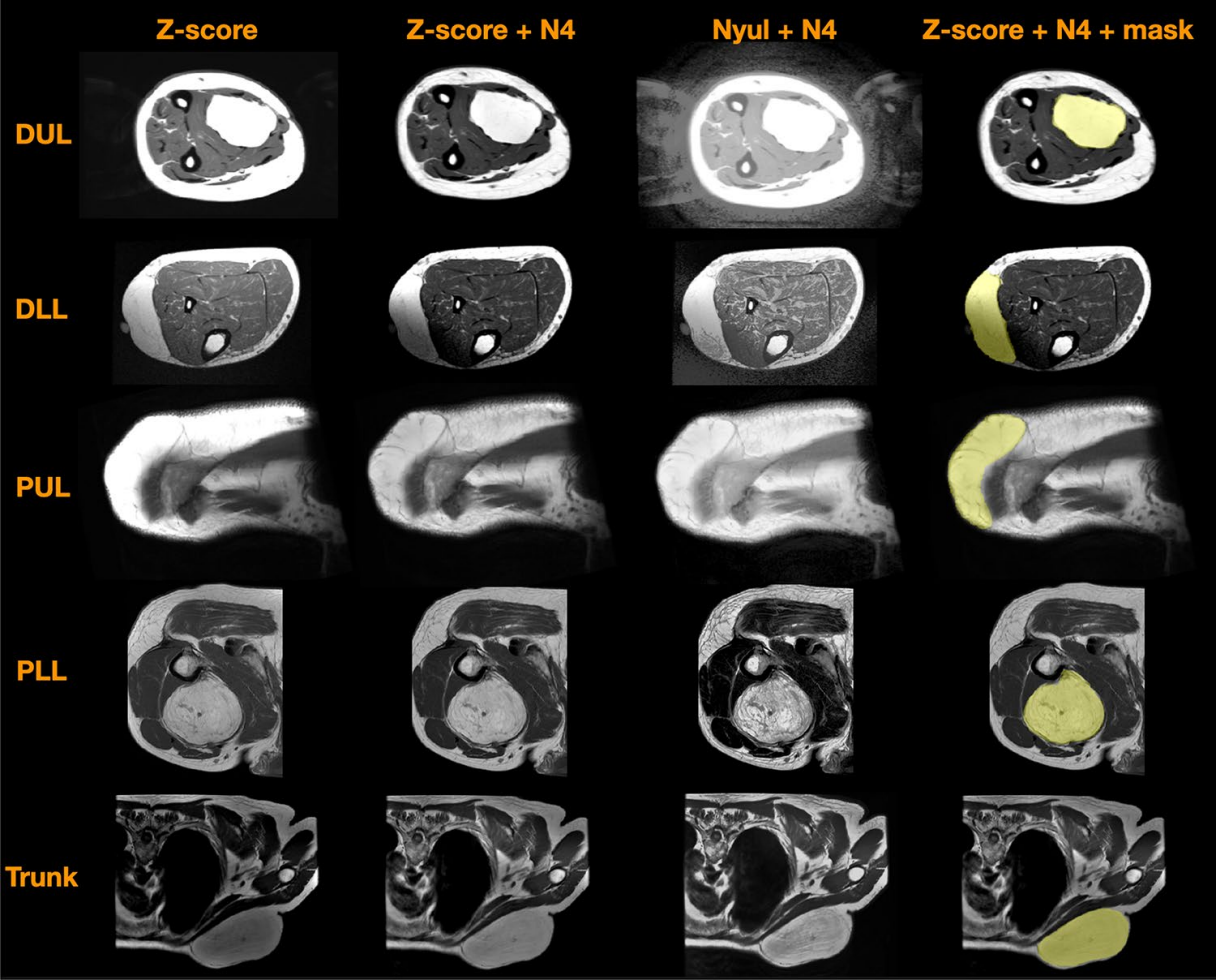
T1-weighted MR images per tumor location (left to right) normalized by Z-score before and after bias correction (N4), normalized by Nyul standardization after N4 correction and the manual delineations (yellow shade) overlaid on MR images. The images are displayed on the same scale. The images (top to bottom) are from distal upper limb (DUL), distal lower limb trunk (DLL), proximal upper limb (PUL), proximal lower limb (PLL), and trunk (T)

Examples of the MR images per tumor location are shown on the same display scale in Fig. 3, with Z-score normalization or Nyul standardization before and after the N4 correction and the corresponding manually delineated tumor masks (yellow). The bias field (or intensity non-uniformity) in the images was mostly corrected using the N4 correction, but it is not always effective because of the data heterogeneity, such as the dimmer area in the lower left of the DLL image in Fig. 3. There are examples without much bias field, such as the PLL case in Fig. 3, that show little changes before and after the N4 correction. The images after the Nyul standardization could show more structural details on the same display scale while enhancing the background, such as the DUL case in Fig. 3.

**Fig. 4:**
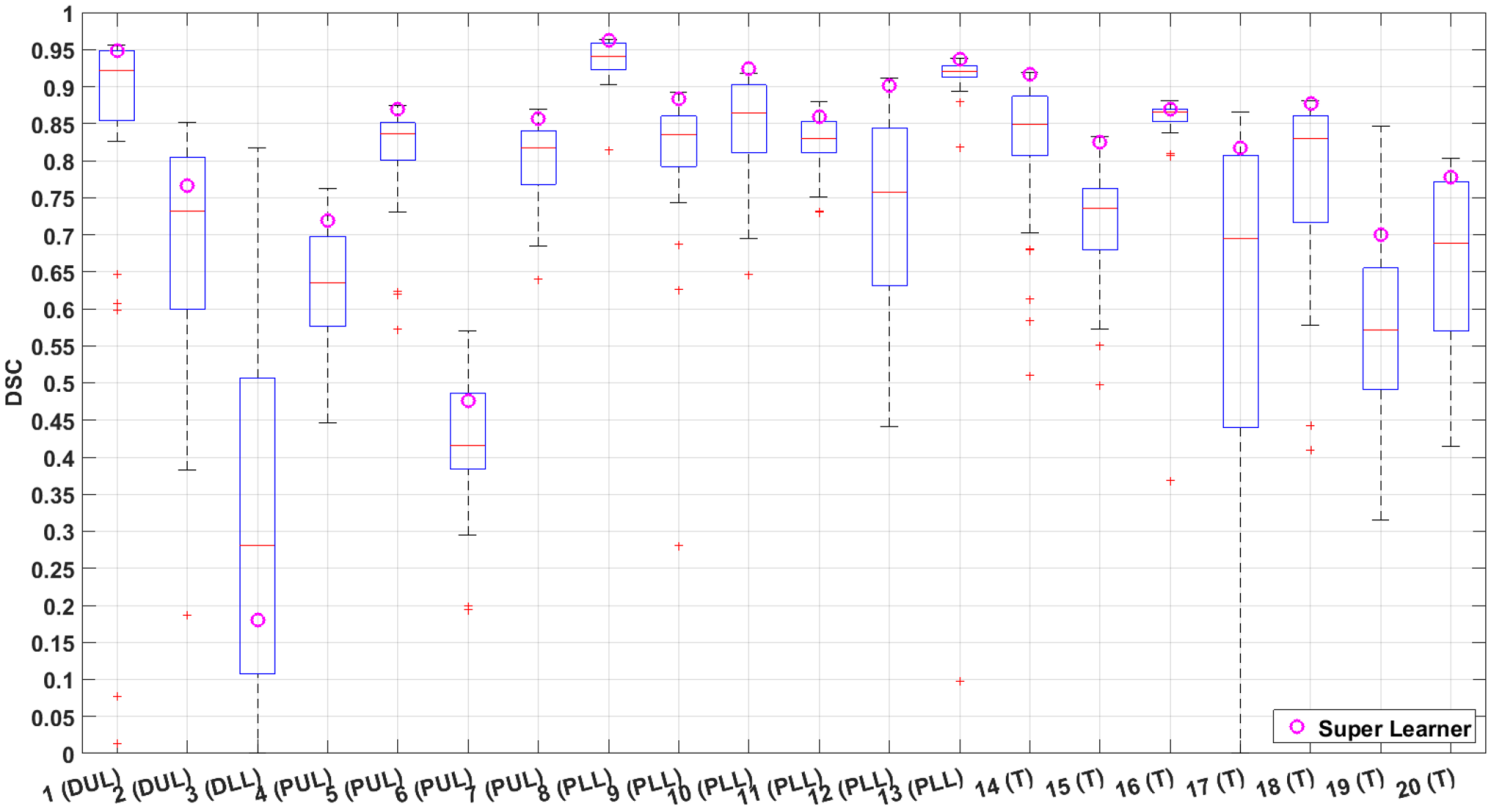
Box plot of dice-similarity-coefficient (DSC) of the testing datasets for the 30 individual base learners and Super Learner (pink circles). Most of Super Learner DSCs are higher than the upper quartile of the individual learners

**Fig. 5:**
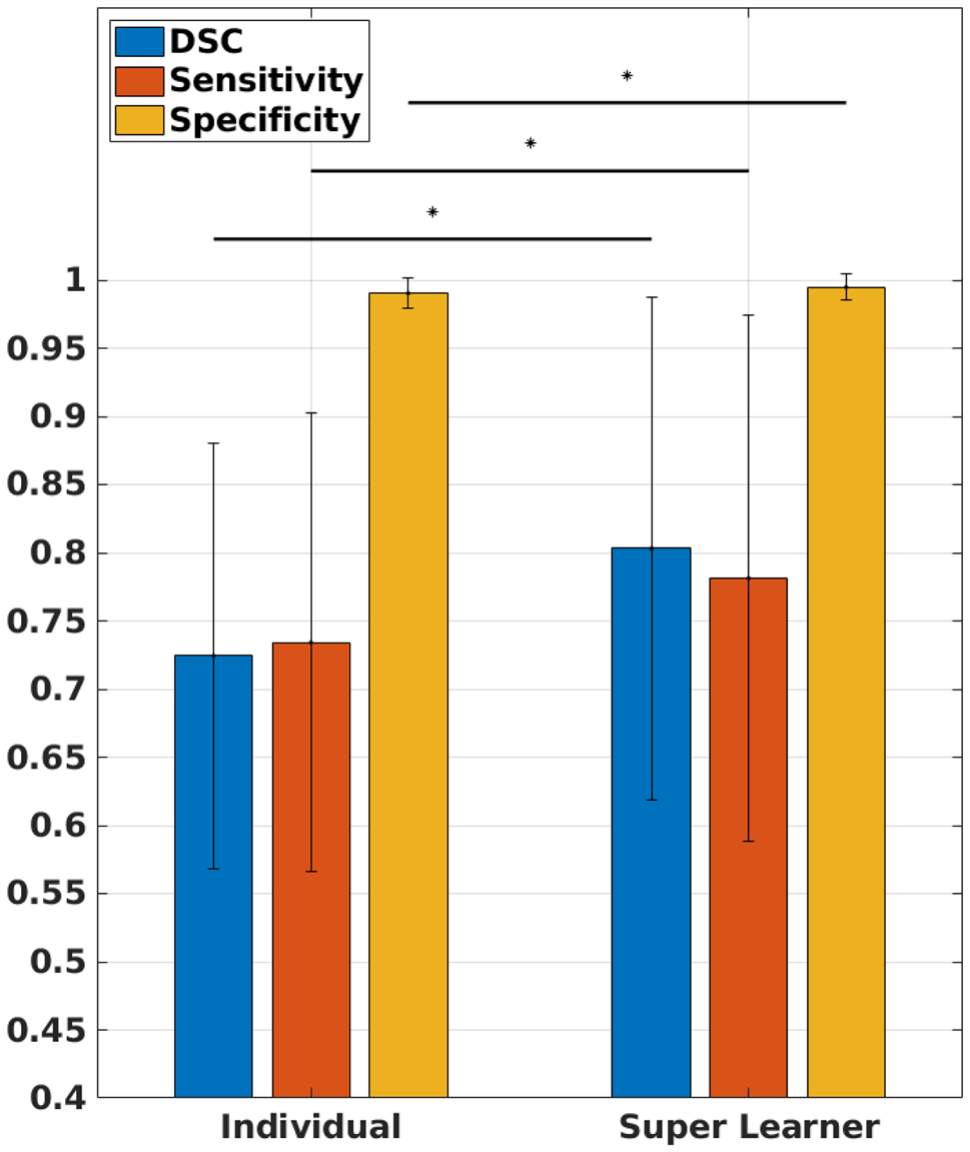
Bar chart of average dice-similarity-coefficient (DSC), sensitivity, and specificity for the individual base learners and Super Learner. The error bars represent the standard deviation calculated across the 20 testing datasets from different tumor locations. ‘*’ indicates the difference is statistically significant (*P* value < 0.05)

**Fig. 6:**
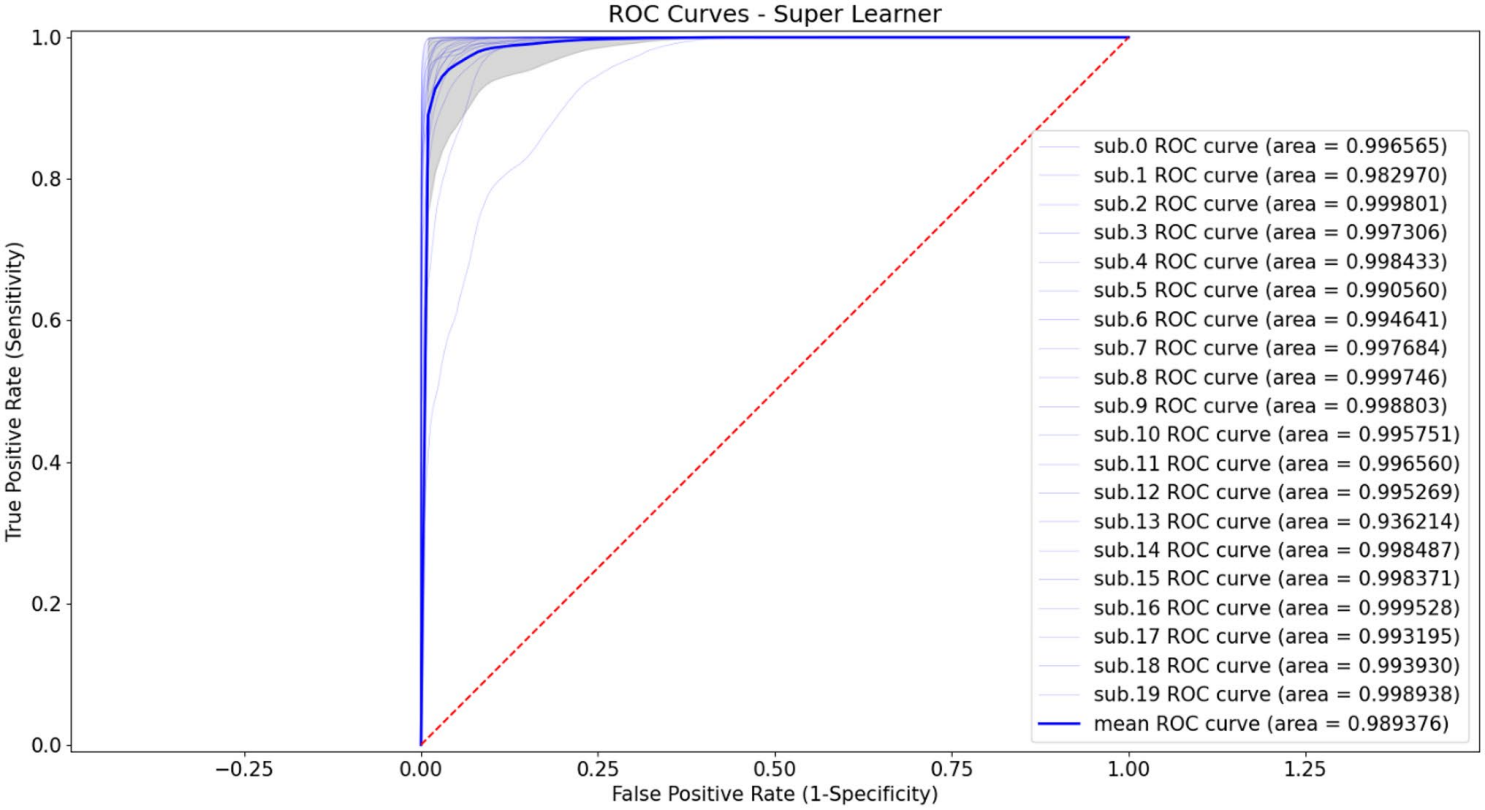
Receiver operating characteristic (ROC) curves and area under curve (AUC) of Super Learner predictions from the 20 testing datasets. The mean ROC curve is in bold blue and the individual curves from the 20 testing datasets are in gray. The gray shadow indicates ± 1 standard deviation

**Fig. 7:**
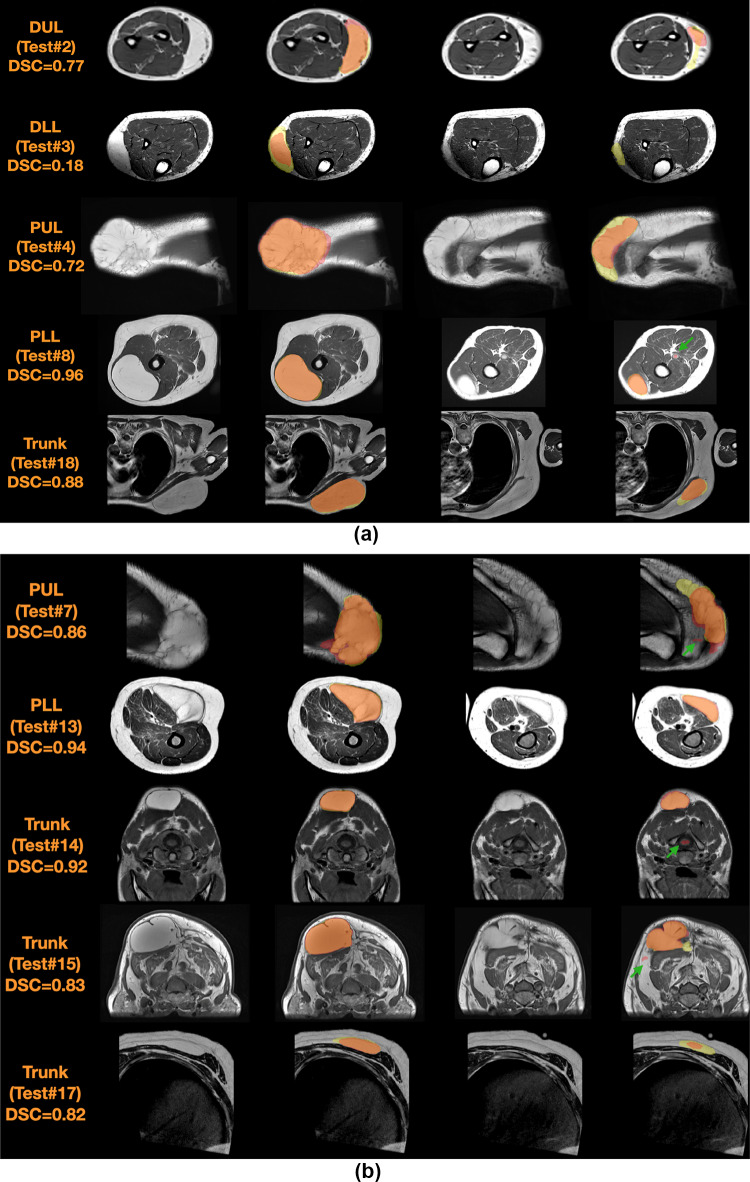
The SL ensemble predictions (red) and tumor delineations (yellow) per tumor location overlaid on the T1-weighted MR images giving orange color for the correctly predicted tumor region. First two columns represent the central image slices of the tumors without and with the delineations and predictions, respectively, whereas the second two columns represent the edge image slices of the tumors. The predictions are often worse in the edge slices than in the central slices of tumor. The dice-similarity-coefficient (DSC) was calculated per volume. Each testing dataset has different voxel size, FOV coverage, and tumor size and shape, but they all have the same matrix size of 512 × 512. The green arrow indicates the misclassification region separated from the tumor. **a** Five representative examples for the tumor locations. **b** Additional five representative examples for the tumor locations of PUL, PLL, and Trunk

The DSCs of the individual base learners per patient exam were box plotted with that of the ensemble predictions in Fig. 4 for the 20 testing data. For the individual base learners, the average DSC, sensitivity, and specificity across all the testing data were 0.724 $\pm$ 0.156, 0.734 $\pm$ 0.168, and 0.990 $\pm$ 0.012, respectively, while for the SL ensemble predictions were 0.803 $\pm$ 0.184, 0.781 $\pm$ 0.193, and 0.995 $\pm$ 0.001. However, two base learners were significantly underperforming; Learner 9 had significantly lower specificity compared to 21 out of the remaining 29 learners (72%); and Learner 30 had significantly lower sensitivity and lower DSC compared to 22 of the remaining 29 learners (76%). To avoid biasing the subsequent comparisons in favor of the SL ensemble predictions, these two underperforming learners were removed for subsequent comparisons, and the average of 28 base learners was compared to SL. The SL ensemble predictions demonstrated significantly higher DSC (0.803 ± 0.184 vs. 0.737 ± 0.157; *P* = 0.0005), sensitivity (0.781 ± 0.193 vs. 0.739 ± 0.173; *P* = 0.001), and specificity (0.995 ± 0.010 vs. 0.991 ± 0.011; *P* < 0.0001). A bar chart of the average results with statistical significance comparisons is shown in Fig. 5. The averages of DSC, sensitivity, specificity, and HD95 per tumor location in the testing data for the individual base learners and SL ensemble predictions as well as their averages of the all 20 testing data are summarized in Table 1. The DSCs, sensitivity, specificity, and HD95 for the individual base learners are summarized in the supplementary Tables S1–S4, respectively. Additionally, the DSCs, sensitivity, specificity, and HD95 of the SLs with optimized weights and equal weights are summarized in the supplementary Table S5. The receiver operating characteristic (ROC) curves of the testing data are plotted in Fig. 6. Five representative examples of the SL ensemble predictions (red) overlaid together with the tumor delineations for the tumor locations on the MR images are shown in Fig. 7a. The additional five examples showing the performance in the tumor locations where the majority of the training data comprises, i.e., PUL, PLL, and Trunk, are presented in Fig. 7b. The predictions of the 30 base learners at the central slice of the tumor are plotted for three patient exams, Test#3 (DLL), 8 (PLL), and 17 (T), in the supplementary Figs. S1–S3 in comparison to SL.

**Table 1:** The averages of DSC, sensitivity, specificity, and 95th percentile Hausdorff Distance (HD95) per tumor location and for all 20-testing data

**Metric**	DUL [2]	DLL [1]	PUL [4]	PLL [6]	T [7]	ALL [20]
**DSC**						
Individual	0.752	0.323	0.665	0.838	0.710	0.724
Super Learner	0.857	0.180	0.730	0.911	0.826	0.803
Improvement	0.105	−0.143	0.065	0.073	0.116	0.079
**Sensitivity**						
Individual	0.784	0.234	0.666	0.802	0.772	0.734
Super Learner	0.806	0.099	0.711	0.862	0.844	0.781
Improvement	0.022	−0.135	0.045	0.060	0.072	0.047
**Specificity**						
Individual	0.996	0.998	0.979	0.995	0.990	0.990
Super Learner	0.999	1.000	0.985	0.999	0.995	0.995
Improvement	0.003	0.002	0.006	0.004	0.005	0.005
**HD95 (mm)**						
Individual	11.5	23.2	25.9	32.1	47.9	34.5
Super Learner	1.7	8.4	15.9	2.2	36.6	15.9
Improvement (%)	85.2	63.8	38.6	93.1	23.6	54.9

## Discussion

The DSC of the predictions for most of the testing data spreads widely between different base learners; this suggests great performance instability when using any single base learner for prediction. Conventionally, only one trained base learner would be used for the final segmentation and could suffer the aforementioned issues due to the data heterogeneity and performance variability. The average DSC, sensitivity, and specificity across all the testing datasets show that the improvement of using SL ensemble framework for prediction is significant (*P* value < 0.05) over those of the individual base learners. The average DSC performance of SL (0.803) from the 20 testing datasets was 10.9% better than that of the individual base learners (0.724). The high specificity resulted from the class imbalance between tumor and non-tumor voxels that is the reason for non-discriminative area under the ROC curve (AUC) between the testing datasets. It is worth noting that the dice-coefficient loss function used for the trainings in this work is insensitive to the class imbalance because it only considers the tumor class but not the background class during trainings, unlike binary cross-entropy function. Given the ROC curves of the testing data, the SL could provide very good discriminant capability between LTs and other tissues on most of the testing data. The threshold was not specifically optimized for every testing dataset, but 0.5 seems to be appropriate for having good sensitivity and specificity in this work.

The major challenge of the deep learning-based LT segmentation in MR images is the data heterogeneity, not only the curated patient exams from multicenter but also the biological characteristics of the LT. In the segmentation of organs or other type of tumors, all the available data would be used for training a neural network without categorizing them for performance evaluation. We introduced a classification method to group the patient exams by the five tumor locations for a systematic analysis. The values and spread of the DSC results from the testing data implied that the segmentation performance may be associated with the tumor location and the individual amount of the training data for each location. Without considering the tumor locations in the trainings, the DSCs of the SL predictions from 17 of the 20 testing data outperformed the 75th percentile of the DSCs from the individual base learners. Only one testing data located in DLL, showed worse DSC of the SL predictions than the median of the individual base learners. To further investigate the relationship between the performance and tumor location, we summarized the average evaluation metrics for each tumor location. All the evaluation metrics were improved when using the SL ensemble learning, except for the DSC and sensitivity in DLL. The lack of patient data in the tumor locations of DLL for training indeed had a negative impact on the tumor segmentation in the same location, while good performance for other tumor locations. We would need more data from each tumor location, especially for DUL and DLL, for training as well as to confirm the performance improvement. In comparison with the testing data number 3 (DLL), number 17 (T) also showed wide spread DSC from the base learners but better performance of SL. The possible reason could be the greater number of patient exams in T than DLL. Although the performance of SL in T was improved for the testing data, the class of trunk may still include distinct variation given the spread of the DSC. This class could be further broken down to different classes.

The HD95 is a spatial distance-based measure and takes into account the difference in contour and location. In addition, the HD95 is more sensitive than other overlap-based measures to any false positive voxel predictions that are spatially located away from the reference standard delineated tumor [35]. The predictions of trunk lesions showed the largest HD95 among all the five tumor locations, because the trunk encompasses a large anatomical area and demonstrates numerous anatomical variations. Additionally, the large FOV coverage is more liable for multiple false positive predictions. In comparison to the individual base learners, the HD95 was reduced substantially by a factor about 23% to 85% (average reduction = 49%) for different tumor locations when using our proposed SL ensemble learning approach.

This study has some limitations. First, the number of datasets per tumor location from all the institutions is still relatively small especially in terms of deep learning training and performance evaluation, although this is by far the largest international multi-institution database of lipomatous tumor that is published. It could cause the class imbalance resulting in prediction bias and decreased the segmentation accuracy for the tumor location of which the training data are fewer. Second, the proposed Super Learner ensemble framework was implemented with 2D neural networks, and the correlation between image slices was not yet considered. Normal tissue could sometimes be misclassified as tumor in nonconsecutive image slices because the information from the neighboring slices is lacking. The disjoint misclassified tumor segments result in the increased HD95 per patient data. Third, the data preprocessing methods used in this work only include basic correction and normalizations. In future work, 3D neural network, more sophisticate data preprocessing methods and classification method of tumor locations will be investigated with the proposed framework to further improve the segmentation accuracy.

## Conclusion

In conclusion, our study demonstrated that the segmentation performance instability caused by the inevitable data heterogeneity and individual trainings could be mitigated using the Super Learner ensemble framework. The whole processing time could be significantly reduced, and the human effort can be mainly focused on reviewing the predictions and correcting any encountered inconsistencies. The predicted tumor segmentation could be used for radiomic analysis and tumor classification. Future work will investigate different base learners other than the correction and normalization methods for the Super Learner ensemble learning and develop state-of-the-art neural network architecture. More institutions have provided data, which are being used for further developing the relationship of the performance and tumor locations. Our future work will validate this tool for lesion classification and characterization, with the ultimate goal to develop a radiologist-friendly tool that can be deployed in clinical practice.

## Supplementary Information

Below is the link to the electronic supplementary material.

Supplementary file1 (DOCX 713 KB)

## Data Availability

Not publicly available.

## Code Availability

Code pertaining to the deep learning model can be made available upon request.
