# Supplementary material for: AI-Based Automated Lipomatous Tumor Segmentation in MR Images: Ensemble Solution to Heterogeneous Data
Source: J Digit Imaging. 2023 Feb 28;36(3):1049–59. doi: 10.1007/s10278-023-00785-1 (PMC10287587; doi:10.1007/s10278-023-00785-1)
Supplement: Supplementary file 1 — Supplementary file1 (DOCX 713 KB) [file 10278_2023_785_MOESM1_ESM.docx]

TABLE S1. Dice-similarity-coefficient (DSC) of each base learner for all the 20 testing datasets

| **DSC** | **Test#1** | **Test#2** | **Test#3** | **Test#4** | **Test#5** | **Test#6** | **Test#7** | **Test#8** | **Test#9** | **Test#10** | **Test#11** | **Test#12** | **Test#13** | **Test#14** | **Test#15** | **Test#16** | **Test#17** | **Test#18** | **Test#19** | **Test#20** |
| --- | --- | --- | --- | --- | --- | --- | --- | --- | --- | --- | --- | --- | --- | --- | --- | --- | --- | --- | --- | --- |
| **Learner 1** | 0.916 | 0.822 | 0.525 | 0.570 | 0.837 | 0.368 | 0.768 | 0.903 | 0.802 | 0.786 | 0.853 | 0.593 | 0.927 | 0.886 | 0.774 | 0.870 | 0.605 | 0.578 | 0.710 | 0.570 |
| **Learner 2** | 0.921 | 0.727 | 0.434 | 0.446 | 0.801 | 0.526 | 0.763 | 0.930 | 0.832 | 0.811 | 0.837 | 0.657 | 0.920 | 0.876 | 0.833 | 0.867 | 0.782 | 0.737 | 0.589 | 0.492 |
| **Learner 3** | 0.925 | 0.821 | 0.108 | 0.630 | 0.874 | 0.475 | 0.753 | 0.916 | 0.687 | 0.647 | 0.730 | 0.441 | 0.914 | 0.889 | 0.749 | 0.873 | 0.440 | 0.587 | 0.529 | 0.415 |
| **Learner 4** | 0.922 | 0.785 | 0.007 | 0.500 | 0.731 | 0.421 | 0.763 | 0.935 | 0.799 | 0.863 | 0.827 | 0.592 | 0.922 | 0.848 | 0.630 | 0.872 | 0.300 | 0.410 | 0.624 | 0.675 |
| **Learner 5** | 0.929 | 0.805 | 0.051 | 0.606 | 0.819 | 0.389 | 0.640 | 0.913 | 0.770 | 0.775 | 0.813 | 0.551 | 0.912 | 0.846 | 0.675 | 0.807 | 0.683 | 0.443 | 0.467 | 0.437 |
| **Learner 6** | 0.903 | 0.713 | 0.187 | 0.632 | 0.837 | 0.405 | 0.818 | 0.961 | 0.839 | 0.858 | 0.853 | 0.911 | 0.918 | 0.807 | 0.809 | 0.877 | 0.836 | 0.703 | 0.788 | 0.531 |
| **Learner 7** | 0.886 | 0.733 | 0.817 | 0.709 | 0.806 | 0.320 | 0.836 | 0.944 | 0.837 | 0.864 | 0.856 | 0.709 | 0.894 | 0.679 | 0.572 | 0.863 | 0.530 | 0.763 | 0.449 | 0.799 |
| **Learner 8** | 0.944 | 0.810 | 0.246 | 0.750 | 0.854 | 0.400 | 0.827 | 0.945 | 0.743 | 0.906 | 0.830 | 0.631 | 0.930 | 0.887 | 0.825 | 0.844 | 0.811 | 0.717 | 0.655 | 0.671 |
| **Learner 9** | 0.598 | 0.788 | 0.435 | 0.646 | 0.573 | 0.302 | 0.744 | 0.815 | 0.626 | 0.695 | 0.853 | 0.517 | 0.818 | 0.510 | 0.497 | 0.809 | 0.332 | 0.709 | 0.353 | 0.672 |
| **Learner 10** | 0.936 | 0.519 | 0.052 | 0.578 | 0.806 | 0.486 | 0.840 | 0.958 | 0.888 | 0.911 | 0.770 | 0.742 | 0.918 | 0.867 | 0.811 | 0.844 | 0.305 | 0.786 | 0.592 | 0.771 |
| **Learner 11** | 0.854 | 0.804 | 0.437 | 0.731 | 0.848 | 0.391 | 0.861 | 0.959 | 0.885 | 0.896 | 0.846 | 0.814 | 0.924 | 0.888 | 0.679 | 0.837 | 0.740 | 0.832 | 0.414 | 0.793 |
| **Learner 12** | 0.826 | 0.808 | 0.295 | 0.707 | 0.820 | 0.411 | 0.857 | 0.961 | 0.837 | 0.902 | 0.867 | 0.868 | 0.930 | 0.871 | 0.763 | 0.850 | 0.859 | 0.829 | 0.592 | 0.785 |
| **Learner 13** | 0.877 | 0.799 | 0.506 | 0.762 | 0.875 | 0.384 | 0.870 | 0.961 | 0.886 | 0.902 | 0.880 | 0.904 | 0.928 | 0.910 | 0.672 | 0.868 | 0.834 | 0.852 | 0.499 | 0.803 |
| **Learner 14** | 0.833 | 0.788 | 0.198 | 0.654 | 0.838 | 0.439 | 0.840 | 0.959 | 0.888 | 0.918 | 0.811 | 0.825 | 0.938 | 0.903 | 0.735 | 0.856 | 0.706 | 0.861 | 0.514 | 0.790 |
| **Learner 15** | 0.867 | 0.851 | 0.158 | 0.712 | 0.851 | 0.410 | 0.854 | 0.961 | 0.827 | 0.908 | 0.847 | 0.864 | 0.926 | 0.849 | 0.801 | 0.857 | 0.838 | 0.865 | 0.538 | 0.790 |
| **Learner 16** | 0.956 | 0.694 | 0.466 | 0.566 | 0.846 | 0.538 | 0.798 | 0.942 | 0.892 | 0.905 | 0.799 | 0.739 | 0.932 | 0.869 | 0.736 | 0.866 | 0.486 | 0.880 | 0.588 | 0.740 |
| **Learner 17** | 0.956 | 0.649 | 0.565 | 0.650 | 0.852 | 0.456 | 0.867 | 0.948 | 0.877 | 0.897 | 0.830 | 0.844 | 0.918 | 0.844 | 0.692 | 0.864 | 0.756 | 0.854 | 0.431 | 0.763 |
| **Learner 18** | 0.952 | 0.518 | 0.652 | 0.619 | 0.851 | 0.516 | 0.799 | 0.940 | 0.862 | 0.881 | 0.752 | 0.714 | 0.913 | 0.836 | 0.716 | 0.853 | 0.770 | 0.866 | 0.683 | 0.624 |
| **Learner 19** | 0.949 | 0.642 | 0.530 | 0.625 | 0.807 | 0.475 | 0.810 | 0.917 | 0.849 | 0.915 | 0.799 | 0.838 | 0.929 | 0.843 | 0.672 | 0.878 | 0.277 | 0.830 | 0.607 | 0.703 |
| **Learner 20** | 0.949 | 0.419 | 0.702 | 0.487 | 0.836 | 0.570 | 0.858 | 0.941 | 0.860 | 0.915 | 0.751 | 0.876 | 0.926 | 0.818 | 0.681 | 0.870 | 0.836 | 0.866 | 0.699 | 0.772 |
| **Learner 21** | 0.954 | 0.811 | 0.110 | 0.619 | 0.854 | 0.557 | 0.830 | 0.922 | 0.813 | 0.865 | 0.851 | 0.772 | 0.936 | 0.882 | 0.742 | 0.869 | 0.866 | 0.854 | 0.491 | 0.740 |
| **Learner 22** | 0.938 | 0.617 | 0.450 | 0.530 | 0.845 | 0.543 | 0.850 | 0.926 | 0.819 | 0.862 | 0.826 | 0.798 | 0.920 | 0.911 | 0.730 | 0.854 | 0.779 | 0.852 | 0.580 | 0.713 |
| **Learner 23** | 0.955 | 0.812 | 0.421 | 0.639 | 0.847 | 0.473 | 0.791 | 0.939 | 0.853 | 0.877 | 0.862 | 0.794 | 0.928 | 0.905 | 0.741 | 0.872 | 0.685 | 0.856 | 0.522 | 0.737 |
| **Learner 24** | 0.954 | 0.599 | 0.000 | 0.576 | 0.857 | 0.307 | 0.826 | 0.933 | 0.822 | 0.863 | 0.816 | 0.631 | 0.915 | 0.919 | 0.686 | 0.861 | 0.720 | 0.747 | 0.562 | 0.611 |
| **Learner 25** | 0.956 | 0.730 | 0.001 | 0.639 | 0.836 | 0.498 | 0.819 | 0.922 | 0.751 | 0.861 | 0.850 | 0.674 | 0.928 | 0.829 | 0.745 | 0.867 | 0.807 | 0.863 | 0.519 | 0.440 |
| **Learner 26** | 0.014 | 0.383 | 0.266 | 0.655 | 0.620 | 0.199 | 0.697 | 0.944 | 0.765 | 0.836 | 0.732 | 0.785 | 0.879 | 0.584 | 0.682 | 0.870 | 0.038 | 0.881 | 0.704 | 0.526 |
| **Learner 27** | 0.647 | 0.437 | 0.099 | 0.659 | 0.746 | 0.295 | 0.815 | 0.940 | 0.792 | 0.702 | 0.820 | 0.512 | 0.893 | 0.703 | 0.780 | 0.866 | 0.000 | 0.779 | 0.846 | 0.632 |
| **Learner 28** | 0.886 | 0.434 | 0.257 | 0.698 | 0.781 | 0.471 | 0.817 | 0.961 | 0.853 | 0.884 | 0.872 | 0.901 | 0.930 | 0.717 | 0.551 | 0.881 | 0.681 | 0.829 | 0.489 | 0.753 |
| **Learner 29** | 0.077 | 0.746 | 0.642 | 0.742 | 0.771 | 0.410 | 0.802 | 0.964 | 0.841 | 0.770 | 0.864 | 0.856 | 0.903 | 0.614 | 0.740 | 0.877 | 0.549 | 0.860 | 0.687 | 0.642 |
| **Learner 30** | 0.607 | 0.186 | 0.069 | 0.572 | 0.623 | 0.194 | 0.685 | 0.923 | 0.281 | 0.749 | 0.813 | 0.585 | 0.097 | 0.681 | 0.751 | 0.369 | 0.000 | 0.582 | 0.315 | 0.499 |
| **mean** | 0.837 | 0.692 | 0.332 | 0.632 | 0.811 | 0.429 | 0.807 | 0.937 | 0.820 | 0.851 | 0.824 | 0.736 | 0.916 | 0.820 | 0.714 | 0.860 | 0.616 | 0.775 | 0.577 | 0.669 |
| **standard deviation (STD)** | 0.235 | 0.142 | 0.234 | 0.079 | 0.069 | 0.089 | 0.053 | 0.029 | 0.062 | 0.072 | 0.042 | 0.133 | 0.023 | 0.106 | 0.080 | 0.018 | 0.246 | 0.126 | 0.114 | 0.121 |
| **STD (%)** | 28.112 | 20.564 | 70.449 | 12.540 | 8.515 | 20.646 | 6.509 | 3.095 | 7.543 | 8.453 | 5.077 | 18.050 | 2.532 | 12.925 | 11.182 | 2.099 | 40.036 | 16.264 | 19.689 | 18.032 |

TABLE S2. Sensitivity of each base learner for all the 20 testing datasets

| **Sensitivity** | **Test#1** | **Test#2** | **Test#3** | **Test#4** | **Test#5** | **Test#6** | **Test#7** | **Test#8** | **Test#9** | **Test#10** | **Test#11** | **Test#12** | **Test#13** | **Test#14** | **Test#15** | **Test#16** | **Test#17** | **Test#18** | **Test#19** | **Test#20** |
| --- | --- | --- | --- | --- | --- | --- | --- | --- | --- | --- | --- | --- | --- | --- | --- | --- | --- | --- | --- | --- |
| **Learner 1** | 0.949 | 0.846 | 0.417 | 0.507 | 0.845 | 0.585 | 0.642 | 0.942 | 0.762 | 0.801 | 0.790 | 0.462 | 0.947 | 0.880 | 0.908 | 0.802 | 0.816 | 0.456 | 0.880 | 0.473 |
| **Learner 2** | 0.949 | 0.694 | 0.284 | 0.341 | 0.826 | 0.560 | 0.638 | 0.940 | 0.804 | 0.816 | 0.762 | 0.526 | 0.945 | 0.920 | 0.946 | 0.799 | 0.724 | 0.664 | 0.979 | 0.382 |
| **Learner 3** | 0.952 | 0.933 | 0.057 | 0.553 | 0.865 | 0.503 | 0.640 | 0.940 | 0.546 | 0.548 | 0.632 | 0.303 | 0.889 | 0.903 | 0.908 | 0.798 | 0.346 | 0.466 | 0.937 | 0.283 |
| **Learner 4** | 0.940 | 0.720 | 0.004 | 0.396 | 0.768 | 0.562 | 0.684 | 0.929 | 0.701 | 0.839 | 0.737 | 0.481 | 0.916 | 0.886 | 0.916 | 0.803 | 0.267 | 0.289 | 0.860 | 0.615 |
| **Learner 5** | 0.946 | 0.967 | 0.029 | 0.513 | 0.848 | 0.444 | 0.539 | 0.937 | 0.745 | 0.753 | 0.766 | 0.426 | 0.912 | 0.854 | 0.929 | 0.704 | 0.637 | 0.312 | 0.844 | 0.320 |
| **Learner 6** | 0.903 | 0.785 | 0.103 | 0.543 | 0.843 | 0.541 | 0.741 | 0.953 | 0.785 | 0.839 | 0.766 | 0.886 | 0.919 | 0.938 | 0.891 | 0.812 | 0.792 | 0.584 | 0.916 | 0.413 |
| **Learner 7** | 0.976 | 0.882 | 0.739 | 0.795 | 0.882 | 0.498 | 0.839 | 0.940 | 0.823 | 0.837 | 0.826 | 0.767 | 0.919 | 0.911 | 0.954 | 0.814 | 0.363 | 0.767 | 0.972 | 0.871 |
| **Learner 8** | 0.919 | 0.965 | 0.140 | 0.722 | 0.809 | 0.436 | 0.760 | 0.941 | 0.627 | 0.866 | 0.761 | 0.474 | 0.929 | 0.880 | 0.942 | 0.747 | 0.716 | 0.616 | 0.935 | 0.585 |
| **Learner 9** | 0.940 | 0.891 | 0.572 | 0.889 | 0.867 | 0.775 | 0.880 | 0.944 | 0.923 | 0.841 | 0.856 | 0.861 | 0.967 | 0.918 | 0.967 | 0.807 | 0.920 | 0.959 | 0.969 | 0.872 |
| **Learner 10** | 0.936 | 0.383 | 0.031 | 0.513 | 0.848 | 0.605 | 0.830 | 0.938 | 0.836 | 0.908 | 0.663 | 0.665 | 0.893 | 0.904 | 0.954 | 0.760 | 0.522 | 0.710 | 0.882 | 0.763 |
| **Learner 11** | 0.961 | 0.812 | 0.282 | 0.709 | 0.842 | 0.497 | 0.805 | 0.937 | 0.828 | 0.871 | 0.743 | 0.729 | 0.931 | 0.924 | 0.968 | 0.765 | 0.619 | 0.795 | 0.963 | 0.841 |
| **Learner 12** | 0.929 | 0.956 | 0.190 | 0.697 | 0.837 | 0.618 | 0.812 | 0.940 | 0.772 | 0.874 | 0.787 | 0.824 | 0.947 | 0.916 | 0.955 | 0.763 | 0.858 | 0.829 | 0.888 | 0.801 |
| **Learner 13** | 0.942 | 0.871 | 0.344 | 0.709 | 0.866 | 0.559 | 0.823 | 0.943 | 0.850 | 0.887 | 0.809 | 0.849 | 0.921 | 0.923 | 0.957 | 0.788 | 0.780 | 0.871 | 0.858 | 0.794 |
| **Learner 14** | 0.944 | 0.881 | 0.110 | 0.609 | 0.857 | 0.517 | 0.789 | 0.938 | 0.851 | 0.895 | 0.715 | 0.745 | 0.933 | 0.924 | 0.959 | 0.791 | 0.579 | 0.820 | 0.938 | 0.806 |
| **Learner 15** | 0.938 | 0.891 | 0.087 | 0.640 | 0.858 | 0.594 | 0.814 | 0.941 | 0.755 | 0.894 | 0.757 | 0.853 | 0.939 | 0.916 | 0.956 | 0.769 | 0.772 | 0.823 | 0.893 | 0.827 |
| **Learner 16** | 0.953 | 0.567 | 0.305 | 0.427 | 0.835 | 0.670 | 0.696 | 0.941 | 0.849 | 0.911 | 0.693 | 0.612 | 0.945 | 0.923 | 0.927 | 0.788 | 0.327 | 0.896 | 0.955 | 0.727 |
| **Learner 17** | 0.950 | 0.511 | 0.396 | 0.542 | 0.796 | 0.648 | 0.804 | 0.941 | 0.867 | 0.887 | 0.730 | 0.811 | 0.954 | 0.920 | 0.967 | 0.783 | 0.633 | 0.847 | 0.947 | 0.803 |
| **Learner 18** | 0.943 | 0.361 | 0.494 | 0.517 | 0.815 | 0.681 | 0.690 | 0.936 | 0.783 | 0.845 | 0.623 | 0.608 | 0.922 | 0.918 | 0.944 | 0.795 | 0.689 | 0.854 | 0.922 | 0.544 |
| **Learner 19** | 0.944 | 0.519 | 0.361 | 0.505 | 0.799 | 0.692 | 0.712 | 0.933 | 0.774 | 0.916 | 0.690 | 0.814 | 0.937 | 0.922 | 0.954 | 0.809 | 0.161 | 0.834 | 0.947 | 0.663 |
| **Learner 20** | 0.940 | 0.276 | 0.542 | 0.359 | 0.810 | 0.723 | 0.814 | 0.940 | 0.813 | 0.929 | 0.633 | 0.818 | 0.952 | 0.912 | 0.898 | 0.793 | 0.789 | 0.861 | 0.888 | 0.821 |
| **Learner 21** | 0.955 | 0.825 | 0.058 | 0.511 | 0.813 | 0.760 | 0.753 | 0.949 | 0.843 | 0.875 | 0.758 | 0.751 | 0.928 | 0.926 | 0.953 | 0.795 | 0.848 | 0.889 | 0.964 | 0.787 |
| **Learner 22** | 0.902 | 0.462 | 0.290 | 0.461 | 0.812 | 0.681 | 0.807 | 0.937 | 0.789 | 0.814 | 0.735 | 0.721 | 0.934 | 0.917 | 0.892 | 0.764 | 0.676 | 0.842 | 0.933 | 0.738 |
| **Learner 23** | 0.953 | 0.792 | 0.267 | 0.558 | 0.810 | 0.571 | 0.680 | 0.940 | 0.808 | 0.867 | 0.801 | 0.686 | 0.918 | 0.919 | 0.860 | 0.796 | 0.621 | 0.854 | 0.933 | 0.773 |
| **Learner 24** | 0.940 | 0.444 | 0.000 | 0.471 | 0.823 | 0.447 | 0.759 | 0.939 | 0.740 | 0.830 | 0.737 | 0.479 | 0.911 | 0.940 | 0.911 | 0.786 | 0.587 | 0.676 | 0.732 | 0.540 |
| **Learner 25** | 0.942 | 0.620 | 0.001 | 0.562 | 0.817 | 0.613 | 0.721 | 0.939 | 0.682 | 0.857 | 0.754 | 0.546 | 0.930 | 0.904 | 0.926 | 0.793 | 0.721 | 0.866 | 0.949 | 0.330 |
| **Learner 26** | 0.007 | 0.608 | 0.188 | 0.551 | 0.557 | 0.199 | 0.571 | 0.939 | 0.724 | 0.785 | 0.636 | 0.684 | 0.800 | 0.750 | 0.787 | 0.823 | 0.048 | 0.839 | 0.745 | 0.406 |
| **Learner 27** | 0.817 | 0.992 | 0.053 | 0.640 | 0.875 | 0.213 | 0.757 | 0.938 | 0.742 | 0.568 | 0.767 | 0.389 | 0.823 | 0.876 | 0.902 | 0.792 | 0.000 | 0.721 | 0.816 | 0.550 |
| **Learner 28** | 0.854 | 0.290 | 0.167 | 0.678 | 0.696 | 0.625 | 0.775 | 0.930 | 0.787 | 0.830 | 0.810 | 0.845 | 0.900 | 0.913 | 0.968 | 0.821 | 0.580 | 0.795 | 0.884 | 0.719 |
| **Learner 29** | 0.043 | 0.685 | 0.482 | 0.687 | 0.723 | 0.553 | 0.753 | 0.936 | 0.739 | 0.691 | 0.803 | 0.777 | 0.843 | 0.828 | 0.949 | 0.828 | 0.501 | 0.842 | 0.974 | 0.590 |
| **Learner 30** | 0.808 | 0.557 | 0.037 | 0.509 | 0.554 | 0.160 | 0.570 | 0.868 | 0.170 | 0.611 | 0.801 | 0.425 | 0.051 | 0.763 | 0.663 | 0.228 | 0.000 | 0.499 | 0.187 | 0.392 |
| **mean** | 0.869 | 0.699 | 0.234 | 0.570 | 0.807 | 0.551 | 0.737 | 0.937 | 0.757 | 0.823 | 0.745 | 0.661 | 0.888 | 0.898 | 0.920 | 0.771 | 0.563 | 0.736 | 0.883 | 0.634 |
| **standard deviation (STD)** | 0.233 | 0.219 | 0.199 | 0.126 | 0.080 | 0.150 | 0.087 | 0.014 | 0.134 | 0.098 | 0.061 | 0.169 | 0.162 | 0.045 | 0.062 | 0.105 | 0.261 | 0.176 | 0.145 | 0.187 |
| **STD (%)** | 26.769 | 31.272 | 84.812 | 22.153 | 9.858 | 27.308 | 11.775 | 1.470 | 17.630 | 11.875 | 8.219 | 25.656 | 18.286 | 5.052 | 6.748 | 13.690 | 46.305 | 23.907 | 16.477 | 29.545 |

TABLE S3. Specificity of each base learner for all the 20 testing datasets

| **Specificity** | **Test#1** | **Test#2** | **Test#3** | **Test#4** | **Test#5** | **Test#6** | **Test#7** | **Test#8** | **Test#9** | **Test#10** | **Test#11** | **Test#12** | **Test#13** | **Test#14** | **Test#15** | **Test#16** | **Test#17** | **Test#18** | **Test#19** | **Test#20** |
| --- | --- | --- | --- | --- | --- | --- | --- | --- | --- | --- | --- | --- | --- | --- | --- | --- | --- | --- | --- | --- |
| **Learner 1** | 0.998 | 0.997 | 0.995 | 0.986 | 0.992 | 0.922 | 0.997 | 0.993 | 0.994 | 0.986 | 0.995 | 0.997 | 0.997 | 0.998 | 0.984 | 0.999 | 0.990 | 0.992 | 0.993 | 0.992 |
| **Learner 2** | 0.998 | 0.997 | 0.999 | 0.990 | 0.989 | 0.972 | 0.997 | 0.996 | 0.995 | 0.989 | 0.995 | 0.998 | 0.997 | 0.997 | 0.988 | 0.998 | 0.999 | 0.991 | 0.984 | 0.993 |
| **Learner 3** | 0.998 | 0.996 | 1.000 | 0.989 | 0.995 | 0.970 | 0.994 | 0.995 | 0.998 | 0.991 | 0.992 | 0.998 | 0.998 | 0.998 | 0.981 | 0.999 | 0.997 | 0.992 | 0.981 | 0.997 |
| **Learner 4** | 0.998 | 0.999 | 0.995 | 0.990 | 0.984 | 0.946 | 0.989 | 0.997 | 0.998 | 0.994 | 0.996 | 0.995 | 0.998 | 0.997 | 0.964 | 0.999 | 0.994 | 0.992 | 0.989 | 0.992 |
| **Learner 5** | 0.999 | 0.995 | 0.997 | 0.990 | 0.989 | 0.959 | 0.985 | 0.994 | 0.992 | 0.989 | 0.990 | 0.996 | 0.997 | 0.997 | 0.970 | 0.999 | 0.997 | 0.994 | 0.979 | 0.994 |
| **Learner 6** | 0.999 | 0.995 | 1.000 | 0.991 | 0.992 | 0.945 | 0.993 | 0.999 | 0.997 | 0.993 | 0.998 | 0.998 | 0.997 | 0.994 | 0.989 | 0.999 | 0.999 | 0.995 | 0.995 | 0.994 |
| **Learner 7** | 0.997 | 0.994 | 0.998 | 0.976 | 0.985 | 0.921 | 0.983 | 0.998 | 0.994 | 0.994 | 0.991 | 0.987 | 0.996 | 0.988 | 0.949 | 0.997 | 1.000 | 0.985 | 0.972 | 0.987 |
| **Learner 8** | 1.000 | 0.995 | 1.000 | 0.989 | 0.996 | 0.964 | 0.992 | 0.998 | 0.998 | 0.997 | 0.994 | 0.999 | 0.998 | 0.998 | 0.987 | 0.999 | 0.999 | 0.994 | 0.989 | 0.994 |
| **Learner 9** | 0.982 | 0.996 | 0.968 | 0.954 | 0.945 | 0.835 | 0.951 | 0.982 | 0.959 | 0.966 | 0.987 | 0.952 | 0.987 | 0.973 | 0.929 | 0.993 | 0.959 | 0.953 | 0.958 | 0.971 |
| **Learner 10** | 0.999 | 0.999 | 0.995 | 0.986 | 0.988 | 0.957 | 0.985 | 0.999 | 0.998 | 0.995 | 0.995 | 0.996 | 0.998 | 0.997 | 0.985 | 0.999 | 0.978 | 0.994 | 0.987 | 0.991 |
| **Learner 11** | 0.996 | 0.997 | 1.000 | 0.988 | 0.993 | 0.949 | 0.993 | 0.999 | 0.998 | 0.996 | 0.999 | 0.998 | 0.997 | 0.998 | 0.968 | 0.998 | 0.999 | 0.993 | 0.968 | 0.989 |
| **Learner 12** | 0.995 | 0.995 | 0.997 | 0.985 | 0.990 | 0.932 | 0.992 | 0.999 | 0.997 | 0.996 | 0.998 | 0.998 | 0.997 | 0.997 | 0.980 | 0.999 | 0.998 | 0.989 | 0.987 | 0.990 |
| **Learner 13** | 0.997 | 0.996 | 1.000 | 0.992 | 0.995 | 0.934 | 0.993 | 0.999 | 0.997 | 0.995 | 0.998 | 0.999 | 0.998 | 0.998 | 0.967 | 0.999 | 0.999 | 0.989 | 0.981 | 0.993 |
| **Learner 14** | 0.995 | 0.996 | 1.000 | 0.986 | 0.991 | 0.959 | 0.991 | 0.999 | 0.997 | 0.997 | 0.996 | 0.998 | 0.998 | 0.998 | 0.976 | 0.998 | 0.999 | 0.995 | 0.980 | 0.990 |
| **Learner 15** | 0.997 | 0.998 | 1.000 | 0.992 | 0.992 | 0.936 | 0.991 | 0.999 | 0.997 | 0.996 | 0.998 | 0.996 | 0.997 | 0.996 | 0.984 | 0.999 | 0.999 | 0.995 | 0.983 | 0.989 |
| **Learner 16** | 0.999 | 0.999 | 1.000 | 0.996 | 0.993 | 0.960 | 0.995 | 0.997 | 0.998 | 0.994 | 0.996 | 0.999 | 0.997 | 0.997 | 0.978 | 0.999 | 1.000 | 0.991 | 0.985 | 0.990 |
| **Learner 17** | 0.999 | 0.999 | 1.000 | 0.993 | 0.997 | 0.942 | 0.995 | 0.998 | 0.996 | 0.995 | 0.998 | 0.996 | 0.996 | 0.996 | 0.970 | 0.999 | 1.000 | 0.991 | 0.971 | 0.988 |
| **Learner 18** | 0.999 | 1.000 | 0.999 | 0.992 | 0.995 | 0.953 | 0.996 | 0.997 | 0.999 | 0.996 | 0.997 | 0.997 | 0.997 | 0.996 | 0.975 | 0.998 | 0.999 | 0.993 | 0.991 | 0.992 |
| **Learner 19** | 0.999 | 0.999 | 1.000 | 0.994 | 0.991 | 0.940 | 0.995 | 0.995 | 0.998 | 0.995 | 0.997 | 0.996 | 0.997 | 0.996 | 0.968 | 0.999 | 1.000 | 0.989 | 0.986 | 0.991 |
| **Learner 20** | 0.999 | 1.000 | 1.000 | 0.994 | 0.994 | 0.960 | 0.992 | 0.997 | 0.997 | 0.994 | 0.996 | 0.998 | 0.997 | 0.995 | 0.973 | 0.999 | 0.999 | 0.992 | 0.992 | 0.988 |
| **Learner 21** | 0.999 | 0.997 | 1.000 | 0.993 | 0.996 | 0.953 | 0.994 | 0.995 | 0.991 | 0.991 | 0.998 | 0.994 | 0.998 | 0.997 | 0.977 | 0.999 | 0.999 | 0.988 | 0.977 | 0.986 |
| **Learner 22** | 1.000 | 1.000 | 1.000 | 0.985 | 0.995 | 0.960 | 0.991 | 0.996 | 0.994 | 0.996 | 0.996 | 0.997 | 0.997 | 0.998 | 0.980 | 0.999 | 0.999 | 0.992 | 0.985 | 0.986 |
| **Learner 23** | 0.999 | 0.998 | 1.000 | 0.990 | 0.995 | 0.959 | 0.996 | 0.997 | 0.997 | 0.994 | 0.995 | 0.999 | 0.998 | 0.998 | 0.983 | 0.999 | 0.998 | 0.991 | 0.980 | 0.987 |
| **Learner 24** | 1.000 | 1.000 | 1.000 | 0.991 | 0.995 | 0.928 | 0.992 | 0.996 | 0.998 | 0.995 | 0.994 | 0.999 | 0.997 | 0.998 | 0.973 | 0.999 | 1.000 | 0.992 | 0.990 | 0.991 |
| **Learner 25** | 1.000 | 0.999 | 1.000 | 0.990 | 0.993 | 0.958 | 0.996 | 0.995 | 0.995 | 0.992 | 0.998 | 0.998 | 0.998 | 0.996 | 0.979 | 0.999 | 0.999 | 0.991 | 0.980 | 0.993 |
| **Learner 26** | 1.000 | 0.981 | 0.993 | 0.993 | 0.989 | 0.961 | 0.993 | 0.998 | 0.993 | 0.995 | 0.991 | 0.998 | 0.999 | 0.987 | 0.981 | 0.998 | 0.983 | 0.996 | 0.996 | 0.994 |
| **Learner 27** | 0.989 | 0.969 | 0.999 | 0.984 | 0.978 | 0.989 | 0.990 | 0.997 | 0.995 | 0.997 | 0.991 | 0.996 | 0.999 | 0.990 | 0.985 | 0.999 | 0.994 | 0.992 | 0.999 | 0.992 |
| **Learner 28** | 0.999 | 0.999 | 0.996 | 0.986 | 0.996 | 0.950 | 0.988 | 1.000 | 0.998 | 0.997 | 0.996 | 0.999 | 0.999 | 0.990 | 0.943 | 0.998 | 0.999 | 0.992 | 0.979 | 0.992 |
| **Learner 29** | 0.999 | 0.998 | 0.999 | 0.991 | 0.993 | 0.944 | 0.987 | 1.000 | 0.999 | 0.994 | 0.995 | 0.999 | 0.999 | 0.986 | 0.977 | 0.998 | 0.996 | 0.993 | 0.990 | 0.990 |
| **Learner 30** | 0.987 | 0.946 | 0.999 | 0.986 | 0.989 | 0.976 | 0.991 | 0.999 | 0.998 | 0.999 | 0.986 | 0.999 | 1.000 | 0.992 | 0.996 | 1.000 | 1.000 | 0.986 | 1.000 | 0.993 |
| **mean** | 0.997 | 0.994 | 0.998 | 0.988 | 0.990 | 0.948 | 0.991 | 0.997 | 0.995 | 0.993 | 0.995 | 0.996 | 0.997 | 0.995 | 0.975 | 0.998 | 0.996 | 0.990 | 0.984 | 0.990 |
| **standard deviation (STD)** | 0.004 | 0.011 | 0.006 | 0.008 | 0.010 | 0.026 | 0.008 | 0.003 | 0.007 | 0.006 | 0.003 | 0.009 | 0.002 | 0.005 | 0.014 | 0.001 | 0.009 | 0.007 | 0.009 | 0.005 |
| **STD (%)** | 0.417 | 1.105 | 0.601 | 0.760 | 0.961 | 2.778 | 0.838 | 0.330 | 0.720 | 0.580 | 0.325 | 0.866 | 0.214 | 0.540 | 1.435 | 0.109 | 0.856 | 0.749 | 0.938 | 0.460 |

TABLE S4. 95th percentile Hausdorff Distance (HD95) of each base learner for all the 20 testing datasets

| **HD95 (mm)** | **Test#1** | **Test#2** | **Test#3** | **Test#4** | **Test#5** | **Test#6** | **Test#7** | **Test#8** | **Test#9** | **Test#10** | **Test#11** | **Test#12** | **Test#13** | **Test#14** | **Test#15** | **Test#16** | **Test#17** | **Test#18** | **Test#19** | **Test#20** |
| --- | --- | --- | --- | --- | --- | --- | --- | --- | --- | --- | --- | --- | --- | --- | --- | --- | --- | --- | --- | --- |
| **Learner 1** | 44.609 | 17.529 | 67.683 | 53.989 | 62.495 | 35.347 | 4.847 | 2.110 | 16.868 | 43.782 | 53.904 | 13.630 | 39.280 | 64.063 | 0.876 | 91.361 | 9.180 | 0.784 | 37.521 | 37.258 |
| **Learner 2** | 45.714 | 12.360 | 39.901 | 56.590 | 60.417 | 31.044 | 4.536 | 2.509 | 14.588 | 30.531 | 57.940 | 8.441 | 81.357 | 46.948 | 1.662 | 92.795 | 3.231 | 0.960 | 37.075 | 40.754 |
| **Learner 3** | 49.231 | 15.586 | 38.074 | 39.462 | 57.187 | 11.690 | 5.981 | 1.959 | 13.959 | 38.195 | 47.967 | 11.417 | 35.876 | 39.269 | 1.616 | 94.554 | 1.176 | 1.108 | 28.626 | 41.295 |
| **Learner 4** | 59.319 | 12.198 | 37.523 | 37.558 | 51.838 | 37.258 | 4.053 | 2.251 | 14.134 | 22.239 | 62.093 | 79.645 | 43.993 | 59.593 | 1.796 | 96.449 | 1.662 | 1.108 | 23.579 | 41.538 |
| **Learner 5** | 48.205 | 9.952 | 71.812 | 48.678 | 54.099 | 37.920 | 13.720 | 3.135 | 23.291 | 47.551 | 57.607 | 8.219 | 41.382 | 54.451 | 1.796 | 95.218 | 1.567 | 11.364 | 29.999 | 80.307 |
| **Learner 6** | 39.695 | 13.670 | 34.109 | 46.069 | 47.641 | 9.866 | 3.159 | 1.662 | 22.884 | 1.239 | 26.363 | 1.616 | 33.967 | 55.120 | 36.044 | 87.012 | 1.959 | 0.876 | 27.552 | 50.226 |
| **Learner 7** | 56.101 | 23.547 | 69.224 | 44.034 | 46.811 | 38.937 | 6.855 | 2.416 | 16.345 | 44.362 | 40.035 | 4.433 | 68.295 | 47.866 | 11.097 | 93.822 | 16.066 | 3.159 | 24.461 | 43.261 |
| **Learner 8** | 29.083 | 12.304 | 32.895 | 39.085 | 46.115 | 6.196 | 4.328 | 2.110 | 0.876 | 8.444 | 40.741 | 2.110 | 30.695 | 43.764 | 2.110 | 89.101 | 1.176 | 1.176 | 21.741 | 42.045 |
| **Learner 9** | 65.534 | 33.124 | 83.701 | 70.874 | 65.702 | 64.365 | 25.872 | 37.617 | 27.221 | 57.223 | 53.321 | 84.505 | 90.925 | 64.077 | 1.998 | 94.123 | 39.322 | 25.835 | 42.269 | 88.956 |
| **Learner 10** | 44.199 | 12.453 | 0.784 | 18.033 | 41.759 | 43.432 | 4.381 | 4.110 | 20.128 | 19.108 | 19.988 | 81.848 | 45.450 | 59.215 | 4.381 | 92.984 | 1.413 | 1.176 | 28.311 | 41.946 |
| **Learner 11** | 46.866 | 9.021 | 0.679 | 9.658 | 18.321 | 3.697 | 3.135 | 5.330 | 21.905 | 29.084 | 44.886 | 2.771 | 48.662 | 54.400 | 1.300 | 94.944 | 2.146 | 0.784 | 29.841 | 42.783 |
| **Learner 12** | 45.887 | 13.532 | 0.554 | 39.378 | 3.799 | 34.718 | 3.060 | 2.658 | 23.380 | 23.540 | 33.029 | 1.176 | 48.279 | 55.672 | 2.110 | 92.140 | 19.046 | 0.784 | 31.277 | 40.790 |
| **Learner 13** | 53.188 | 3.255 | 0.679 | 35.979 | 13.961 | 4.702 | 2.771 | 1.998 | 19.888 | 1.176 | 9.046 | 1.239 | 49.419 | 55.677 | 1.662 | 92.572 | 13.103 | 0.960 | 31.606 | 2.384 |
| **Learner 14** | 48.331 | 10.587 | 0.876 | 16.340 | 16.648 | 7.992 | 4.165 | 3.159 | 23.391 | 15.599 | 14.161 | 2.509 | 42.751 | 50.929 | 2.110 | 91.833 | 1.176 | 0.876 | 30.367 | 49.641 |
| **Learner 15** | 43.119 | 5.003 | 0.679 | 43.650 | 40.012 | 23.915 | 3.918 | 1.998 | 23.268 | 25.152 | 19.596 | 1.413 | 37.093 | 57.715 | 1.176 | 94.812 | 2.110 | 1.300 | 29.783 | 61.801 |
| **Learner 16** | 54.672 | 8.918 | 38.656 | 40.619 | 58.276 | 5.825 | 3.634 | 1.998 | 0.784 | 7.699 | 8.630 | 4.972 | 43.399 | 48.213 | 1.616 | 93.974 | 1.662 | 1.108 | 32.693 | 44.571 |
| **Learner 17** | 62.706 | 5.994 | 37.107 | 47.884 | 39.747 | 2.743 | 3.371 | 2.318 | 0.784 | 27.071 | 3.135 | 2.879 | 50.175 | 57.666 | 2.629 | 94.231 | 5.981 | 0.784 | 28.624 | 55.878 |
| **Learner 18** | 50.278 | 18.553 | 43.532 | 22.086 | 51.859 | 8.303 | 4.310 | 32.060 | 0.784 | 16.128 | 7.445 | 6.318 | 29.827 | 48.446 | 3.371 | 93.237 | 2.416 | 1.959 | 31.156 | 58.157 |
| **Learner 19** | 50.926 | 10.313 | 39.980 | 40.874 | 37.332 | 6.045 | 4.310 | 1.959 | 0.876 | 27.524 | 8.935 | 5.574 | 84.061 | 56.007 | 2.416 | 93.426 | 1.413 | 0.784 | 33.374 | 42.582 |
| **Learner 20** | 55.675 | 16.902 | 42.265 | 42.205 | 51.594 | 5.094 | 5.094 | 1.959 | 0.784 | 8.413 | 5.891 | 1.176 | 54.052 | 48.869 | 3.996 | 94.297 | 1.959 | 0.784 | 33.967 | 38.632 |
| **Learner 21** | 53.083 | 20.179 | 43.438 | 73.943 | 56.022 | 16.566 | 3.231 | 2.285 | 0.784 | 39.584 | 3.348 | 1.108 | 62.701 | 51.526 | 1.616 | 95.174 | 0.960 | 1.616 | 35.364 | 25.325 |
| **Learner 22** | 52.998 | 27.165 | 76.618 | 65.371 | 41.310 | 7.064 | 4.310 | 2.110 | 0.876 | 20.387 | 48.224 | 1.998 | 56.909 | 50.548 | 2.743 | 94.614 | 2.036 | 0.554 | 35.624 | 35.497 |
| **Learner 23** | 58.418 | 26.809 | 39.380 | 50.881 | 48.756 | 4.398 | 4.328 | 1.959 | 0.784 | 8.268 | 41.198 | 80.721 | 47.731 | 49.100 | 0.876 | 95.564 | 1.176 | 0.960 | 34.991 | 36.752 |
| **Learner 24** | 48.603 | 15.849 | 37.170 | 37.709 | 42.905 | 82.897 | 6.032 | 2.351 | 0.784 | 9.156 | 4.914 | 1.616 | 45.252 | 53.557 | 2.110 | 95.904 | 1.300 | 0.784 | 38.211 | 0.784 |
| **Learner 25** | 51.157 | 23.540 | 42.603 | 64.221 | 51.894 | 26.803 | 2.879 | 1.959 | 0.679 | 17.453 | 6.843 | 1.567 | 73.774 | 54.127 | 1.662 | 95.047 | 1.176 | 0.784 | 30.817 | 37.950 |
| **Learner 26** | 39.736 | 10.233 | 30.114 | 23.952 | 48.873 | 46.483 | 5.094 | 1.708 | 16.919 | 19.231 | 65.428 | 73.709 | 15.196 | 56.742 | 44.832 | 84.561 | 1.998 | 3.918 | 15.693 | 46.979 |
| **Learner 27** | 36.686 | 16.890 | 31.277 | 37.932 | 36.673 | 30.149 | 5.917 | 1.959 | 24.748 | 17.919 | 62.641 | 86.076 | 22.571 | 39.534 | 38.529 | 0.784 | 1.567 | 3.060 | 22.649 | 52.573 |
| **Learner 28** | 55.959 | 13.709 | 0.679 | 3.527 | 1.662 | 38.725 | 3.548 | 1.662 | 10.760 | 1.176 | 54.266 | 75.678 | 40.793 | 50.471 | 4.398 | 91.146 | 0.960 | 3.899 | 18.747 | 42.466 |
| **Learner 29** | 42.350 | 14.134 | 0.554 | 2.351 | 43.418 | 23.725 | 3.527 | 1.959 | 20.338 | 1.959 | 14.942 | 100.189 | 46.577 | 55.949 | 1.616 | 44.089 | 2.509 | 3.085 | 16.061 | 48.332 |
| **Learner 30** | 31.587 | 16.085 | 1.176 | 30.466 | 2.318 | 30.040 | 5.213 | 5.583 | 24.339 | 20.797 | 61.129 | 0.000 | 29.470 | 35.755 | 39.310 | 5.315 | 9.566 | 8.486 | 14.461 | 52.847 |
| **mean** | 48.797 | 14.980 | 32.791 | 39.447 | 41.315 | 24.198 | 5.319 | 4.628 | 12.905 | 21.666 | 32.588 | 24.952 | 47.997 | 52.176 | 7.448 | 85.503 | 5.034 | 2.827 | 29.215 | 44.144 |
| **standard deviation (STD)** | 8.433 | 6.781 | 25.271 | 18.136 | 18.011 | 19.748 | 4.366 | 8.299 | 10.023 | 14.840 | 22.221 | 35.837 | 17.697 | 6.740 | 13.045 | 24.275 | 8.014 | 4.956 | 6.963 | 17.100 |
| **STD (%)** | 17.282 | 45.267 | 77.068 | 45.977 | 43.594 | 81.608 | 82.072 | 179.313 | 77.669 | 68.496 | 68.186 | 143.626 | 36.872 | 12.918 | 175.144 | 28.390 | 159.208 | 175.310 | 23.834 | 38.738 |

TABLE S5. The SL performance comparison between optimized weights (ensemble) and equal weights (unweighted)

|  |  | **Test#1** | **Test#2** | **Test#3** | **Test#4** | **Test#5** | **Test#6** | **Test#7** | **Test#8** | **Test#9** | **Test#10** | **Test#11** | **Test#12** | **Test#13** | **Test#14** | **Test#15** | **Test#16** | **Test#17** | **Test#18** | **Test#19** | **Test#20** |
| --- | --- | --- | --- | --- | --- | --- | --- | --- | --- | --- | --- | --- | --- | --- | --- | --- | --- | --- | --- | --- | --- |
| DSC | ensemble | 0.948 | 0.767 | **0.180** | **0.719** | 0.869 | 0.476 | **0.857** | **0.963** | **0.884** | **0.924** | **0.859** | **0.901** | **0.937** | 0.917 | 0.825 | **0.869** | **0.817** | **0.877** | 0.700 | **0.777** |
|  | unweighted | 0.948 | **0.771** | 0.081 | 0.681 | **0.878** | **0.517** | 0.828 | 0.956 | 0.861 | 0.894 | 0.841 | 0.750 | 0.934 | **0.922** | **0.850** | 0.859 | 0.720 | 0.829 | **0.726** | 0.697 |
| Recall | ensemble | **0.930** | **0.681** | **0.099** | **0.616** | **0.843** | **0.591** | **0.792** | **0.940** | **0.812** | **0.891** | **0.764** | **0.834** | **0.930** | **0.922** | **0.962** | **0.792** | **0.722** | **0.825** | **0.931** | **0.752** |
|  | unweighted | 0.918 | 0.672 | 0.042 | 0.549 | 0.810 | 0.523 | 0.721 | 0.925 | 0.769 | 0.825 | 0.730 | 0.602 | 0.902 | 0.889 | 0.937 | 0.768 | 0.570 | 0.748 | 0.903 | 0.594 |
| Specificity | ensemble | 1.000 | 0.999 | 1.000 | 0.995 | 0.995 | 0.956 | 0.994 | 0.999 | 0.999 | 0.998 | 0.999 | 0.999 | 0.998 | 0.999 | 0.986 | 0.999 | 0.999 | 0.997 | 0.991 | 0.993 |
|  | unweighted | 1.000 | 0.999 | 1.000 | **0.997** | **0.998** | **0.975** | **0.998** | **1.000** | 0.999 | 0.999 | 0.999 | **1.000** | 0.999 | 0.999 | **0.990** | 0.999 | **1.000** | 0.996 | **0.993** | **0.995** |
| HD95 (mm) | ensemble | 0.8 | 2.6 | **8.4** | **9.1** | 8.0 | 45.9 | 0.8 | **0.8** | **4.7** | **1.4** | **3.6** | **1.8** | 1.4 | 0.8 | 42.0 | **2.6** | **2.5** | 37.3 | 111.9 | 31.3 |
|  | unweighted | 0.8 | **2.3** | 14.0 | 11.7 | **1.7** | **41.8** | **0.5** | 1.2 | 6.1 | 1.6 | 3.9 | 4.6 | **1.1** | **0.4** | **40.0** | 3.0 | 6.8 | **37.0** | **111.1** | **31.0** |


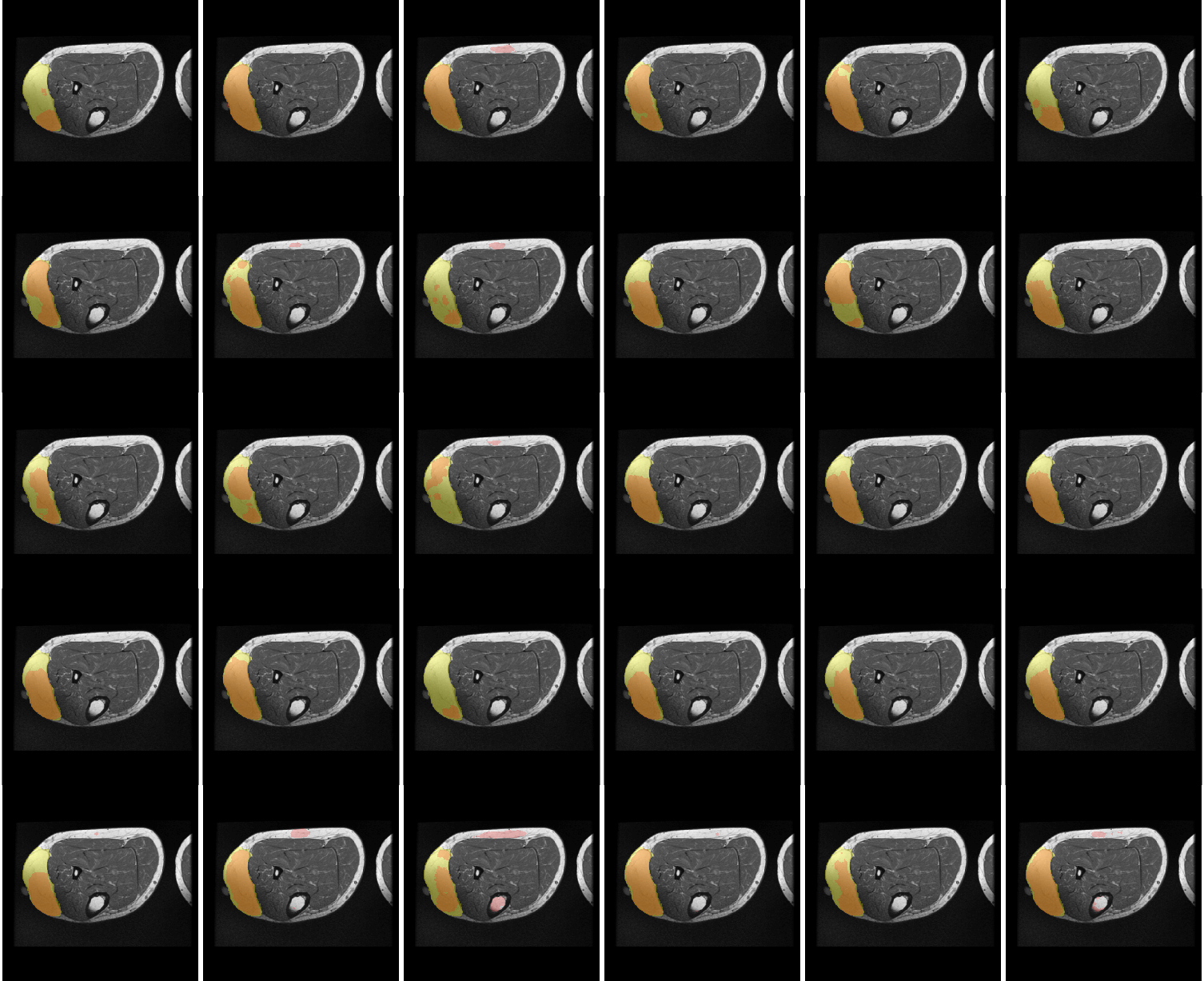


Figure S1. The base leaner predictions (red) and tumor delineations (yellow) at the central slice of the tumor for Test#3 (DLL), the exam with the worst SL performance.


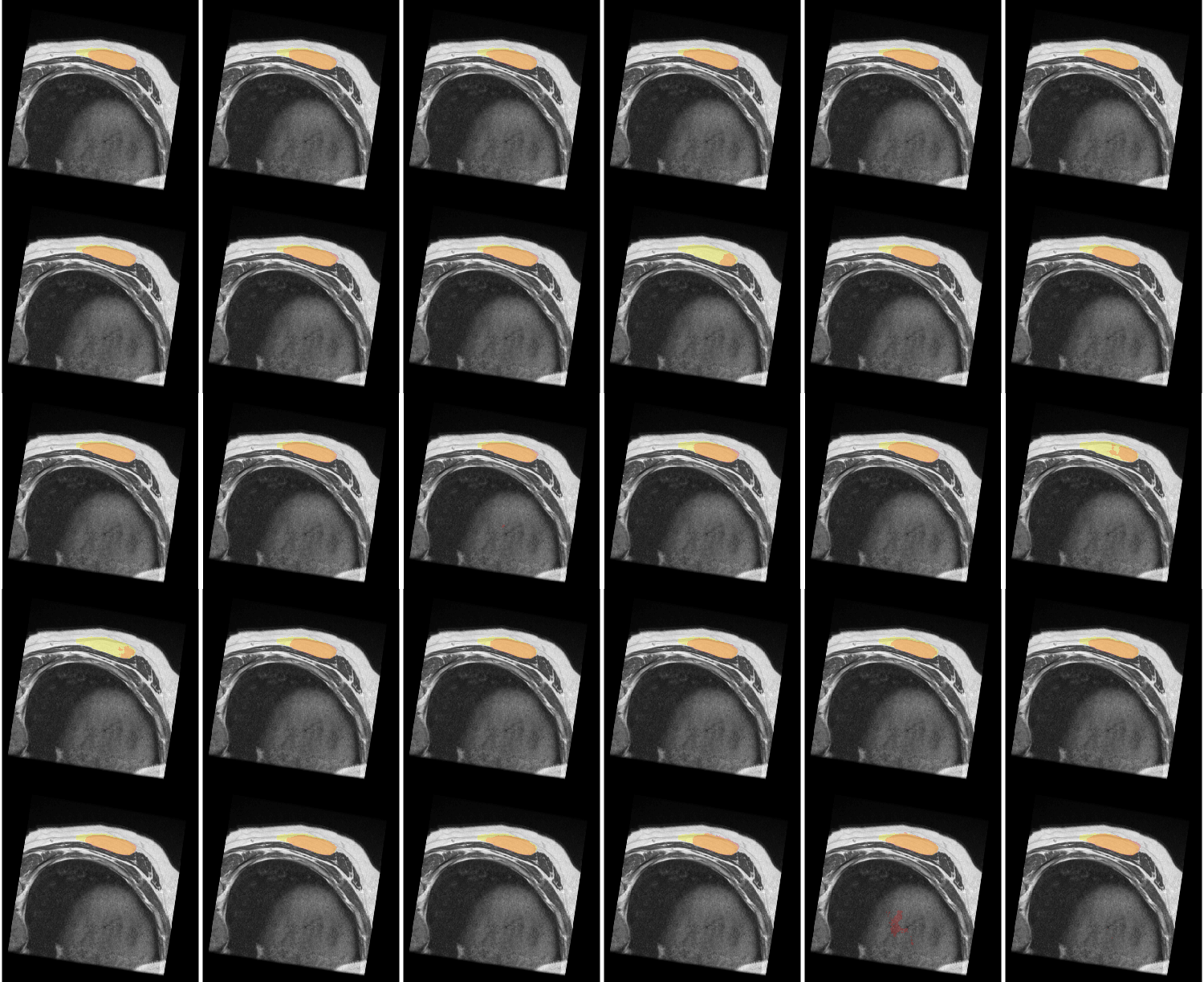


Figure S2. The base leaner predictions (red) and tumor delineations (yellow) at the central slice of the tumor for Test#17 (T), the exam with widest spreading DSCs.


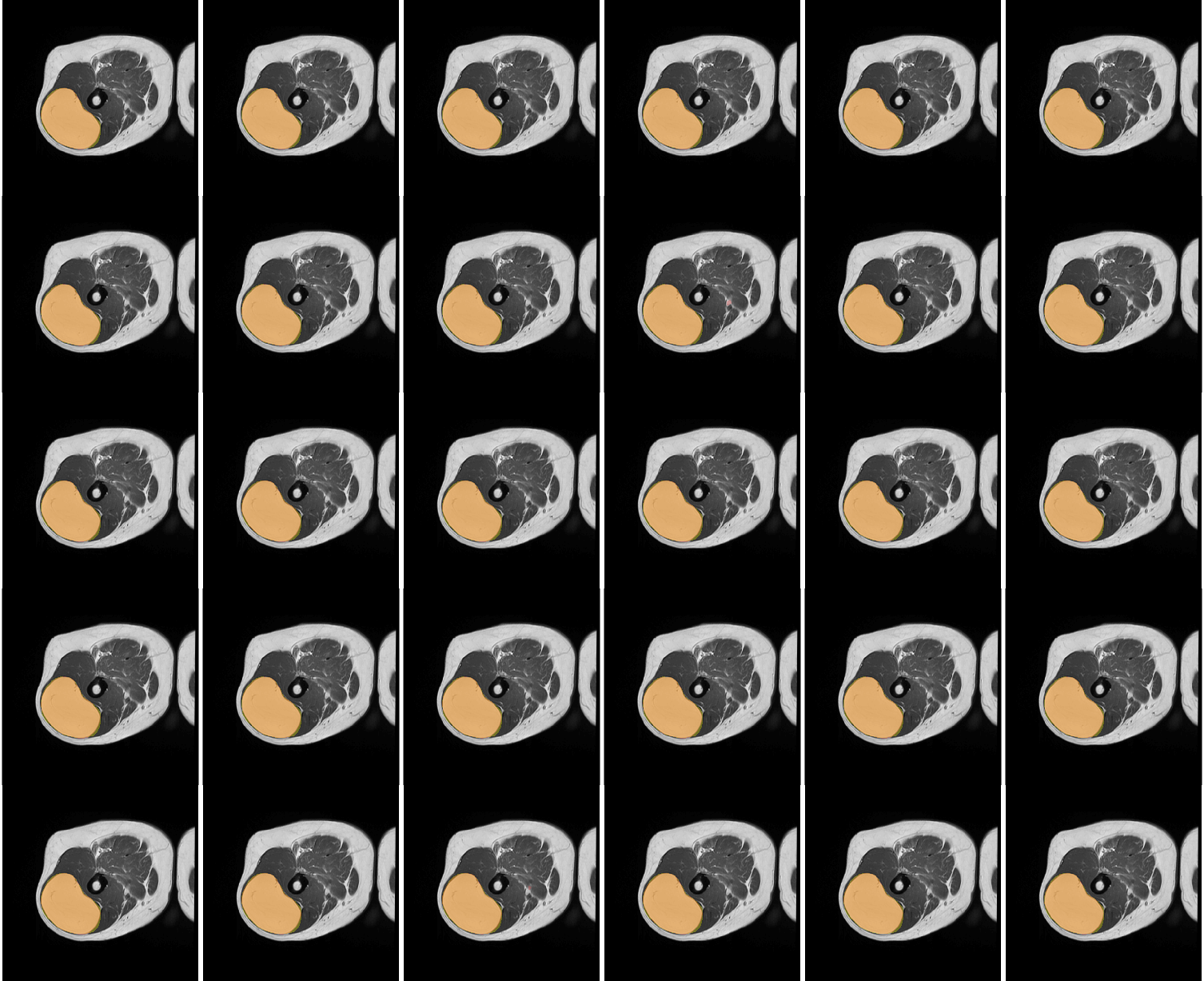


Figure S3. The base leaner predictions (red) and tumor delineations (yellow) at the central slice of the tumor for Test#8 (PLL), the exam with the best SL performance.
